# Liquid Metal–Polyphenol Hybrids for Solar Steam Generation

**DOI:** 10.1002/advs.202512789

**Published:** 2025-10-13

**Authors:** Nieves Flores, Franco Centurion, Md. Hasan Al Banna, Nur‐Adania Nor‐Azman, Moonika. S. Widjajana, Yuqin Wang, Li Liu, Shih‐Hao Chiu, Majharul Haque Khan, Sarina Sarina, Mohammad B Ghasemian, Francois‐Marie Allioux, Kourosh Kalantar‐Zadeh, Md. Arifur Rahim

**Affiliations:** ^1^ School of Chemical and Biomolecular Engineering University of Sydney Sydney NSW 2006 Australia; ^2^ School of Chemical Engineering University of New South Wales (UNSW) Sydney NSW 2052 Australia; ^3^ Sydney Analytical University of Sydney Sydney NSW 2006 Australia; ^4^ Department of Chemical and Biological Engineering Monash University Clayton VIC 3800 Australia

**Keywords:** desalination, gallium, graphene, liquid metal, metal‐phenolic gel, MPNs, solar steam generation

## Abstract

Interfacial solar steam generation has emerged as a promising strategy for sustainable water desalination; however, achieving high efficiency under practical conditions remains a significant challenge. Here, a natural polyphenol‐based gel composite incorporating liquid gallium particles and graphene is presented, engineered for high‐performance solar‐driven desalination. This synergistic combination enables broadband light absorption, heat localization, and rapid water transport. Graphene and the polyphenol gel matrix enhance light absorption and solar‐to‐heat conversion, while liquid gallium serves to localize heat; the gel structure facilitates water retention and vapor escape. These features are critical for maximizing solar energy utilization and sustaining continuous water evaporation under real‐world conditions. Under one sun irradiation, the system achieves an evaporation rate of 4.8 kg m^−2^ h^−1^ for deionized water and 3.4 kg m^−2^ h^−1^ for seawater. These findings highlight the potential of such multifunctional gel composites in addressing global freshwater scarcity through scalable and energy‐efficient desalination technologies.

## Graphical abstract

The combination of liquid gallium nanoparticles, graphene, and a metal‐phenolic gel creates a unique architecture that can act as a highly efficient broad–spectrum solar light absorber. The resulting composite applied for solar steam generation can produce clean water with an evaporation rate of 3.4 kg m^−2^ h^−1^ under 1 Sun illumination from seawater.

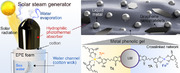

## 1 Introduction

Access to clean and potable water remains a critical global challenge, particularly in arid and water‐scarce regions.^[^ ^1^, ^2^, ^3^ ^]^ Conventional desalination technologies, such as reverse osmosis^[^ ^4^ ^]^ and multi‐stage flash distillation, are highly energy‐intensive and thus challenging for widespread implementation.^[^ ^5^ ^]^ In contrast, interfacial solar steam generation (ISSG) has recently emerged as a sustainable and low‐energy solution to water purification.^[^ ^6^, ^7^, ^8^, ^9^, ^10^ ^]^ This approach employs light‐absorbing materials that float on the surface of water and convert the absorbed sunlight into heat, driving water evaporation at the air‐liquid interface.^[^ ^3^ ^]^ However, the efficiency of ISSG systems is often limited by thermal losses,^[^ ^11^ ^]^ inadequate heat localization, salt accumulation on the solar absorber surface,^[^ ^12^, ^13^ ^]^ and inefficient water transport.

Photothermal materials used for solar harvesting are designed to convert absorbed sunlight into heat, thereby increasing the system temperature.^[^ ^14^, ^15^, ^16^ ^]^ However, efficiently harvesting the full solar spectrum while minimizing heat losses remains a challenge.^[^ ^14^, ^17^, ^18^ ^]^ In this regard, ideal solar absorbers should achieve high absorption, low emission, and suitable heat conduction.^[^ ^15^ ^]^ So far, the solar thermal energy conversion mechanisms have primarily been relied on the localized plasmonic effect of noble metal nanoparticles,^[^ ^19^ ^]^ nonradiative relaxation in semiconductors,^[^ ^20^ ^]^ and molecular thermal vibrations in carbon‐based materials.^[^ ^21^ ^]^ Hybrid photothermal materials have also gained interest due to their composite structures, which offer synergistic enhancements in photothermal conversion efficiency.^[^ ^22^, ^23^ ^]^

Liquid metal (LM), such as gallium (Ga) and its alloys, are promising candidate for photothermal applications in nano‐dimensional structures,^[^ ^24^ ^]^ due to their electromagnetic absorption within visible^[^ ^25^, ^26^, ^27^ ^]^ and near‐infrared (NIR) regions^[^ ^28^ ^]^ and high thermal conductivity,^[^ ^29^, ^30^ ^]^ facilitating efficient heat transfer.^[^ ^31^ ^]^ They also exhibit potential for use as thermal interface materials.^[^ ^32^, ^33^, ^34^, ^35^, ^36^, ^37^, ^38^ ^]^ However, the oxidation susceptibility of liquid metal NPs in an aqueous environment necessitates stabilization strategies to maintain their functional properties.

A notable strategy for stabilizing and functionalizing LM particles involves the use of ligand molecules.^[^ ^39^, ^40^, ^41^, ^42^, ^43^, ^44^, ^45^ ^]^ In this context, a practical approach may involve the use of natural polyphenols as ligands that both protect LM particles and form a cross‐linked network, capable of enhancing light absorption, structural integrity, thermal insulation, and water transport, all of which are essential for efficient solar desalination.^[^ ^40^, ^46^ ^]^ Among the large library of natural polyphenols, tannic acid (TA) —rich in catechol and gallol groups, is known to form surface‐confined films and bulk gels through coordination‐driven assembly with various transition metals.^[^ ^47^ ^]^ Polyphenols are natural ultraviolet (UV) light absorbers, and their coordination with transition metals can form a range of coloured complexes (e.g., TA/ Fe^+3^ complexes are dark blue),^[^ ^48^ ^]^ extending their light absorbance from UV to visible and NIR regions.^[^ ^49^ ^]^ Notably, TA can produce bulk gel networks, known as metal‐phenolic gels, with group IV transition metal ions such as titanium ions.^[^ ^50^, ^51^, ^52^ ^]^ Such gel structures can provide hydrophilic networks^[^ ^51^ ^]^ with low thermal conductivity, facilitating heat retention^[^ ^53^ ^]^ and efficient water transport. Recently, we reported a photothermal absorber composite of LM Ga particles and a polyphenol‐based coordination ink for thermoelectricity generation. The polyphenol ink enabled broad light absorption, while Ga enhanced thermal and electrical performance.^[^ ^53^ ^]^ However, the previous system did not fully exploit synergistic interactions with other light‐absorbing or conductive components, nor did it leverage structural flexibility to support water management functionalities—both essential for expanding its applicability to solar‐driven water desalination. This direction is particularly promising in the context of global water scarcity, where efficient, scalable solar thermal evaporators are urgently needed.

In this study, we develop a photothermal composite system by integrating graphene, LM Ga particles into, a metal‐phenolic gel (MPG) structure composed of TA, iron, and titanium (TA–Fe^3+^–Ti^4+^). This composite material maximizes solar absorption, improves heat management, and optimizes water evaporation rates. The LM component enhances heat transfer and photothermal absorption in visible and IR regions, while the MPG facilitates a more efficient and broad‐band solar absorption, water transport, to some extent salt rejection,^[^ ^49^ ^]^ ligand‐to‐metal charge‐transfer (LMCT),^[^ ^51^ ^]^ surface wetting properties, and vapor release. Graphene was also incorporated to further contribute to light‐to‐heat conversion efficiency^[^ ^54^ ^]^ and thermal conductivity,^[^ ^55^ ^]^ reinforcing the synergistic interactions between these materials. Through systematic evaluation of surface temperature, evaporation rates under simulated solar irradiation, and salt rejection performance, we demonstrate the potential of this composite system for high‐efficiency seawater purification.

## 2 Results and Discussion

### 2.1 Preparation and Characterization of the LM‐Graphene‐MPG Composite

**Figure 1:**
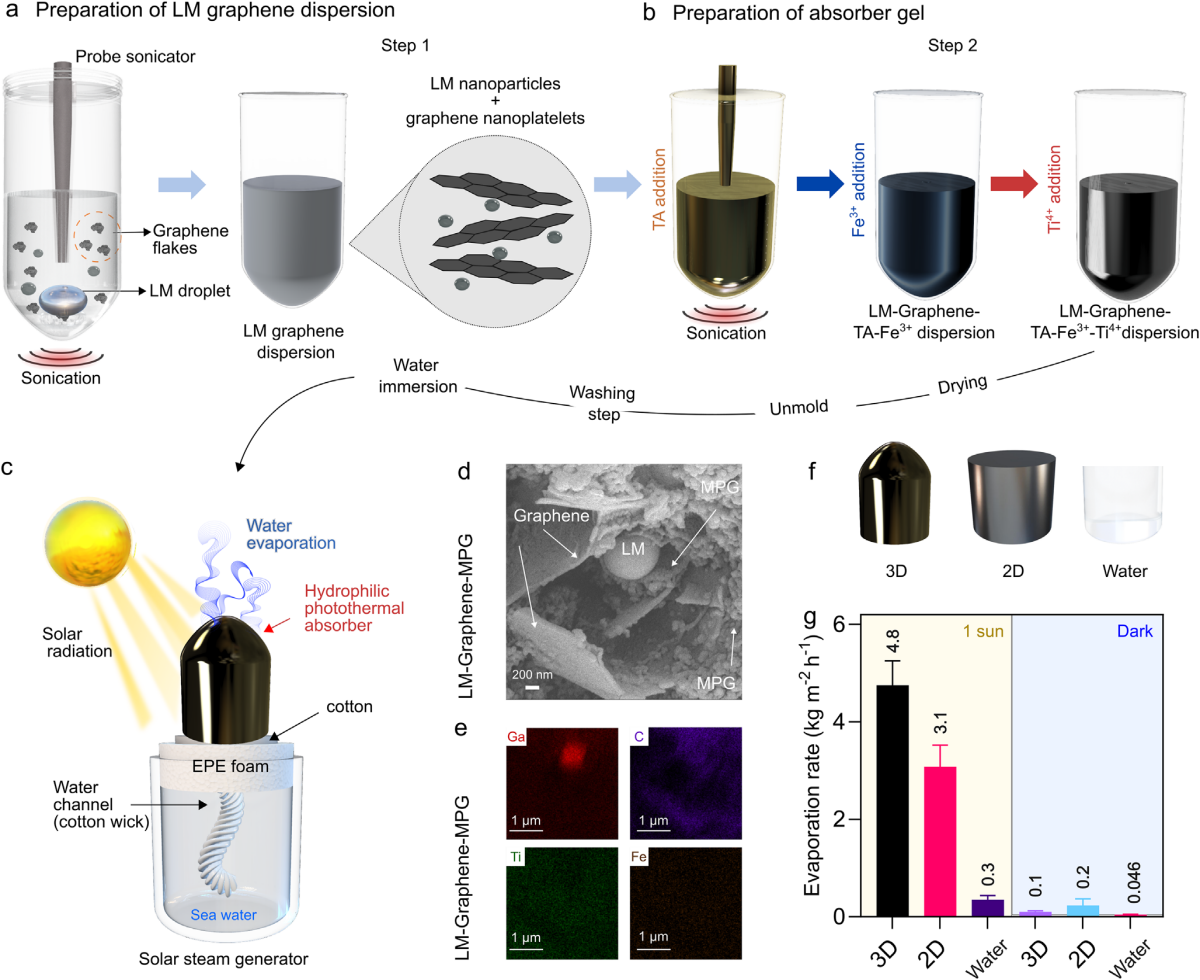
Schematic diagram for preparation of LM–Graphene–MPG. a) Preparation of LM–Graphene dispersion. b) Preparation of LM–Graphene–MPG. c) Solar steam generator. d) SEM images of LM–Graphene–MPG. e) EDS analyses of LM–Graphene–MPG f) 3D shape, 2D shape, water. g) Evaporation rate of pure water under 1 Sun.

The photothermal composite developed in this study was fabricated through a two‐step process, as illustrated in **Figure** **1**. First, large LM droplets and graphene nanoplatelets were subjected to high‐power sonication (Figure 1a) in isopropanol to produce a LM and graphene dispersion (LM particle size distribution ranged from 459 to 1990 nm with a mean particle size of ≈ 1100 nm). This resulting dispersion was then sonicated again in the presence of TA. Here, the TA molecules were self‐assembled and cross‐linked with the LM droplets and graphene, creating a core–shell configuration in which LM served as the core. Since TA is known for its antioxidant properties,^[^ ^56^ ^]^ its assembly on the LM surface effectively minimized oxidants^[^ ^41^ ^]^ and free radicals^[^ ^57^ ^]^ during sonication, thus reducing oxide layer formation on the LM particles.^[^ ^41^ ^]^ Second, this dispersion containing LM, graphene, and TA, was used to form a gel structure (MPG) by complexing TA with Fe^3+^ and Ti^4+^ ions (Figure 1b). In this step, TA present in the dispersion was sequentially cross‐linked with Fe^3+^and Ti^4+^ precursors to establish the gel matrix with dark colour.^[^ ^53^ ^]^ As a result, the LM–Graphene–MPG contained two distinct coordination complexes (TA–Fe^3+^ and TA–Ti^4+^) capable of absorbing visible light across multiple wavelengths (Figure S1, Supporting Information) originating from their distinct LMCT bands.^[^ ^48^, ^51^ ^]^ Additionally, the presence of graphene, which exhibits low reflection loss, further enhanced the broad‐spectrum solar absorption.^[^ ^58^ ^]^

Next, a typical solar steam generator model was constructed using the composite (Figure 1c). In this setup, the LM–Graphene–MPG absorbs and converts sunlight to heat while providing a broad surface area for water evaporation. The composite was moulded into a 3D conical structure, a geometry previously reported to induce a non‐uniform temperature gradient across the evaporator surface, thereby enhancing the evaporation rate.^[^ ^59^ ^]^ Moreover, the 3D evaporator increases the effective surface area for energy harvesting.^[^ ^60^ ^]^ To retain heat and prevent the gel from submerging in the bulk water, an expanded polyethylene (EPE) foam layer was placed beneath it. The EPE is a closed‐pore, hydrophobic thermal insulator with a thermal conductivity of 0.032 W m^−1^ K^−1^.^[^ ^61^ ^]^ A cotton wick was threaded through the foam to transport water from the bulk reservoir to the gel. Additionally, a cotton layer was incorporated between the EPE foam and the gel to enhance water distribution.

Considering the different components used in the photothermal device, we performed structural and morphological characterization of the LM–Graphene–MPG gels using a range of spectroscopic and microscopic techniques. Transmission electron microscopy (TEM) images revealed a core–shell structure with TA assembled on the LM surface (Figure S2, Supporting Information). Scanning electron microscopy (SEM) coupled with energy dispersive X‐ray spectroscopy (EDS) was also performed to probe the elemental mapping of C, O, Ti, and Fe showed a homogeneous distribution of these elements on the surface of LM particles (Figure 1e, Figure S2–S5, Supporting Information).

**Figure 2:**
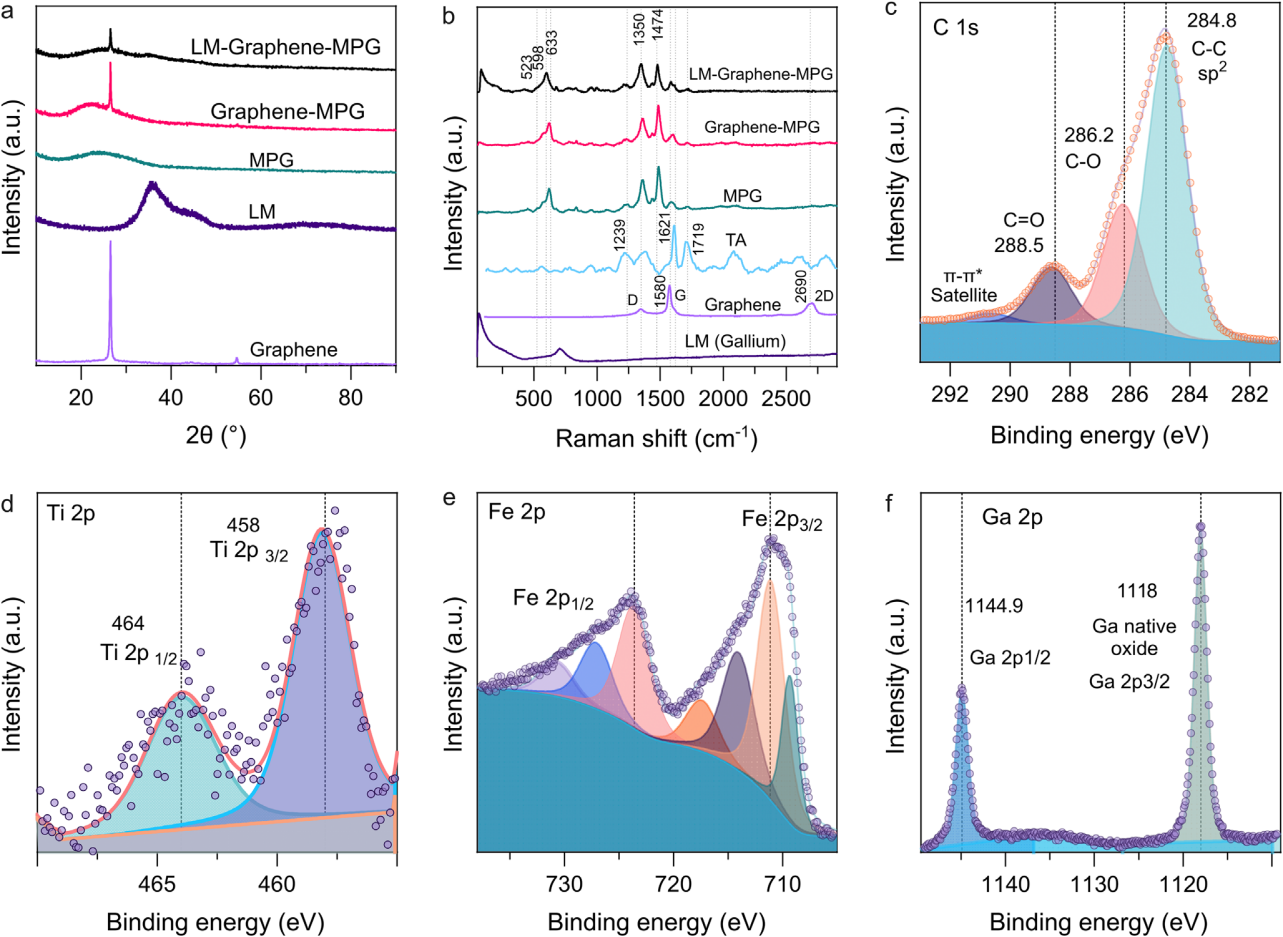
Structural and chemical characterization of composite samples a) XRD patterns of LM–Graphene–MPG, graphene, and LM. b) Raman spectra of MPG samples, TA, graphene, and LM. c–f) XPS spectra of LM–Graphene–MPG, including C 1s, Ti 2p, Fe 2p, and Ga 2p, core level spectra, showing the chemical composition of the composites.

We examined the crystallinity of MPG, Graphene–MPG, and LM–Graphene–MPG gels using X‐ray diffraction (XRD) analysis. For the sample LM–Graphene–MPG, the patterns (**Figure** **2**a) revealed a prominent graphene peak at 26.4°, corresponding to the (003) crystal faces of graphite (PDF # 560159), and a Ga peak at 35.8°, indicative of Ga in its liquid state.^[^ ^62^, ^63^, ^64^ ^]^ Additionally, an amorphous band between 16° and 30° was observed, which can be attributed to the asymmetric structure of the TA– Fe^3+^– Ti^4+^ complex.

To further elucidate the molecular interactions within these systems, we employed Raman spectroscopy (Figure 2b), which provided complementary insights into the composition and coordination environment of the MPG gels. The Raman spectra of LM–Graphene–MPG, Graphene–MPG, and MPG exhibited similar profiles, with the characteristic graphene G band at 1580 cm^−1^ overlapping with signals from the TA–Fe^3+^–Ti^4+^ complex. Additional peaks at ≈606 cm^−1^ indicated the chelation of Fe^3+^ by phenolic oxygen in the TA^[^ ^65^ ^]^ confirming the formation of metal‐ligand coordination bonds. This coordination is closely associated with the enhanced red‐NIR photon absorption due to the LMCT effect, facilitated by the abundant TA–Fe and TA–Ti complexes. Furthermore, dominant bands at ≈1344 and ≈1472 cm^−1^, attributed to ring stretching coupled to (C–O) and (C–H) vibrations (with a downshift is observed upon increasing the metal concentration (Ti and Fe)),^[^ ^66^, ^67^ ^]^ affirmed the presence of the TA complex. The latter band, linked to benzene vibrations coupled to C–O stretching and C–H bending, suggests a significant contribution to carbon‐oxygen stretching interactions. This complexation results in an enhanced Raman response due to strong polarizability variations, further supporting the role of titanium coordination in stabilizing the MPG gels and promoting their effective photothermal performance.

X‐ray photoelectron spectroscopy (XPS) was carried out to investigate the chemical composition of the LM–Graphene–MPG. In the C1s XPS spectrum, the peaks revealed the presence of sp^2^ C–C (284.8 eV), C–O (286.4 eV), O–C = O (288.9 eV), and π–π* shake‐up satellite peaks (291 eV), respectively, consistent with the structure of TA (Figure 2c). The presence of two characteristic peaks at binding energy (BE) of ≈464 and ≈458 eV,^[^ ^68^ ^]^ indicating the chemical states of Ti 2*p*_1/2_ and Ti 2*p*_3/2_, respectively (Figure 2d), corroborates the existence of the 4+ oxidation state of Ti ions in the MPG, as also reported elsewhere.^[^ ^69^ ^]^ Besides, the presence of Fe^3+^ was also identified by XPS (Figure 2e) in the MPG with the two major peaks at BE of ≈711 and ≈725 eV that can be attributed to Fe 2*p*_3/2_ and Fe 2*p*_1/2_, respectively.^[^ ^70^ ^]^ Additionally, metal ion chelation caused a shift in the phenolic oxygen peak from 529.4 eV to a higher BE of 531.2 eV in the O1*s* spectra (Figure S3, Supporting Information), indicating the electron transfer process from TA to the metal ions.^[^ ^71^ ^]^ From the XPS analysis of the LM (Ga) component in the composite, the Ga 3*d* and Ga 2*p* spectra confirmed the presence of Ga in the LM–Graphene–MPG. Furthermore, the Ga 3*d* spectrum (Figure S6, Supporting Information) showed the presence of Ga^3+^ species at BE of ≈20.7 eV^[^ ^72^ ^]^ and also showed the presence of the elemental Ga^0^ (≈18.8 eV).^[^ ^43^, ^73^, ^74^ ^]^ The Ga 2p spectrum further confirms these assignments, where the BE of ≈1118.51 eV can be attributed to Ga^3+^ species^[^ ^75^, ^76^, ^77^, ^78^ ^]^ that originate from the thin oxide layer formed during the sonication (Figure 2f). Note that additional XPS Spectra from MPG, graphene nanoplatelets, and TA are shown in Figure S7–S9 (Supporting Information) for reference.

Water transport is a critical factor for efficient solar desalination, which is relevant to the hydrophilicity of the materials. To evaluate this property, we measured the water contact angles of MPG, Graphene–MPG, and LM–Graphene–MPG. The MPG exhibited a contact angle of ≈28.5 ± 3.2°, indicating excellent hydrophilicity and rapid water absorption, with droplets fully permeating the surface within seconds. This can be attributed to the strong hydrogen bonding between TA and water, which enhances water transport through the gel matrix.^[^ ^79^ ^]^ In comparison, Graphene–MPG and LM–Graphene–MPG showed higher contact angles of 55.6 ± 2.2° and 62.5 ± 0.2°, respectively (Figure S10, Supporting Information), reflecting moderately hydrophilic behaviour.

Thermogravimetric analysis (TGA) was used to assess the thermal stability of the composites. Aside from water loss, the LM–Graphene–MPG exhibited 12% weight loss between 40–169 °C, indicating stability within this temperature range. As shown in Figure S11 (Supporting Information), under N_2_ atmosphere the TG curves of TA and MPG display two major mass loss events. TA begins to decompose at 230–350 °C (due to outer‐layer gallic acid units decarboxylation),^[^ ^80^ ^]^ and above 350 °C (inner‐layer decomposition and char formation). For the MPG, when TA is coordinated with Fe^3+^ and Ti^4+^, the decomposition started earlier ≈169 °C as shown in Figure S12 (Supporting Information), but the material exhibited enhanced stability above 500 °C, resulting in substantially higher char residue due to metal–polyphenol coordination. At 500 °C, the residues were 33.3% (TA), 38.3% (MPG), 58.9% (LM–Graphene–MPG), 61.1% (Graphene–MPG), 95.1% (graphene), and 100%. (LM), underscoring the role of graphene and LM in enhancing thermal resistance.

### 2.2 Photothermal Properties of the Composite

**Figure 3:**
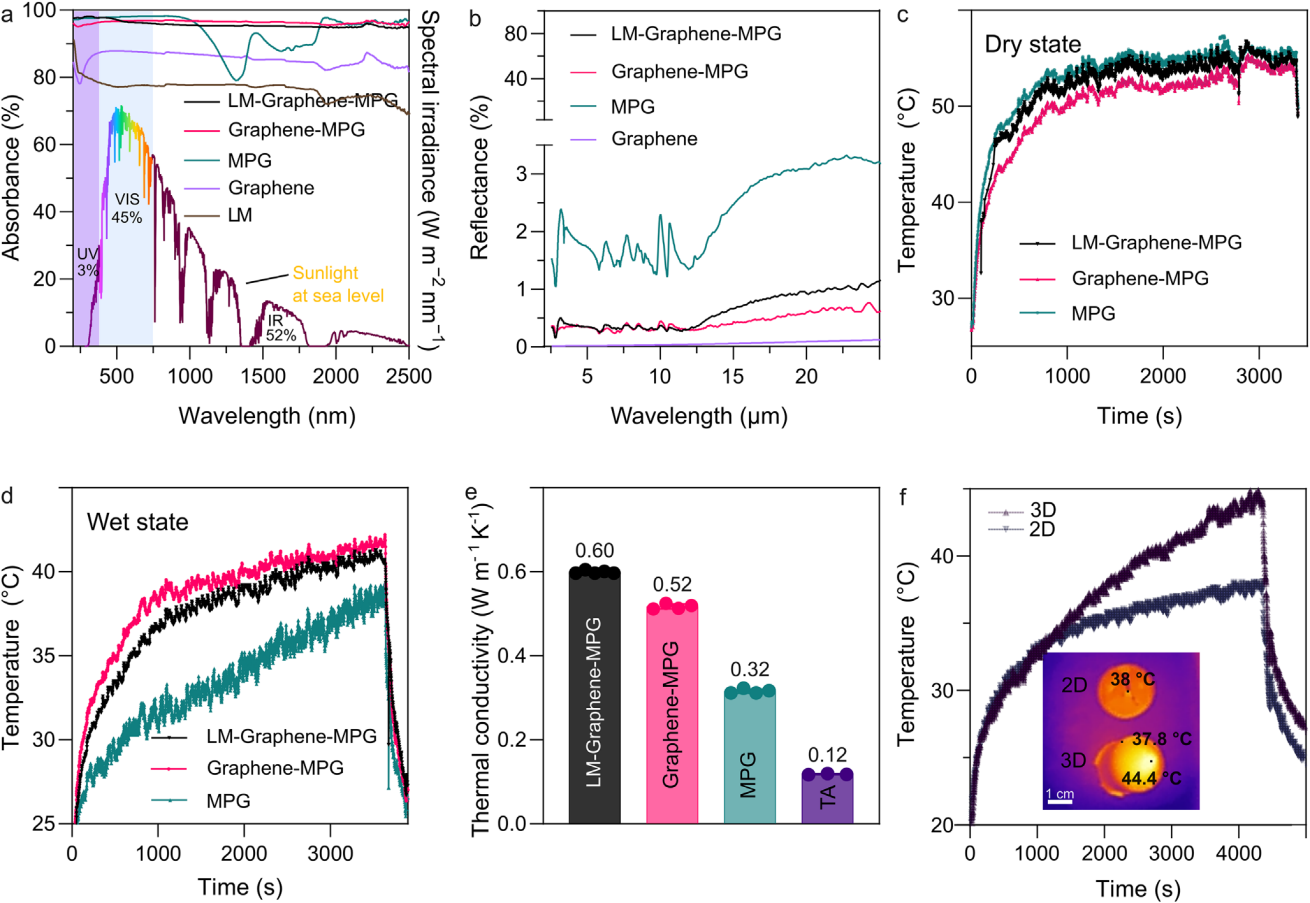
Photothermal performance of the composites. a) Absorbance spectra of the LM–Graphene–MPG, Graphene–MPG, MPG, graphene, and LM in the 250–2500 nm wavelength range, Solar spectrum at sea level. b) Reflectance of LM–Graphene–MPG, Graphene–MPG, and MPG samples in the 2.0–25 µm band. c) Temperature profiles of LM–Graphene–MPG, Graphene–MPG, MPG relative to heating time under 1 Sun. d) Temperature profiles of LM–Graphene–MPG, Graphene–MPG, and MPG samples in wet conditions. e) Thermal conductivity of LM–Graphene–MPG, Graphene–MPG, MPG, TA. f) Temperature profile of 2D and 3D LM–Graphene–MPG in dry state, inset shows the IR thermal image.

Broadband absorption of solar light is essential to develop efficient photothermal materials, as solar irradiance spans ultraviolet, visible, and NIR wavelengths. To assess this capacity, UV‐VIS‐NIR spectra were recorded (**Figure** **3**a). The MPG gel achieved ≈79% absorption in the 600–1060 nm range. This performance is attributed to LMCT bands within the conjugated TA–Fe^3+^–Ti^4+^ composite,^[^ ^81^, ^82^ ^]^ where metal ion chelation by polyphenols increases photon absorption and lowers the material's bandgap, enhancing its light harvesting capacity.^[^ ^83^ ^]^ When graphene and Ga were incorporated, overall absorption rose to ≈95% over the 200–2500 nm range, a value surpassing many existing photothermal materials and underscoring its potential for high‐efficiency vapor generation.^[^ ^84^, ^85^, ^86^, ^87^ ^]^

Reflectance measurements (Figure 3b) further highlight the strong light‐trapping characteristics of Graphene–MPG and LM–Graphene–MPG, where both exhibit minimal reflectance (≈1%) across the mid‐IR spectral range (2.5–25 µm). Given that solar absorbers emit thermal radiation across mid‐IR spectrum to the environment while absorbing solar radiation across UV‐NIR spectrum.^[^ ^88^ ^]^ Due to the low reflectance, some re‐radiation in the mid‐IR region is observed,^[^ ^89^ ^]^ which can limit heat retention under solar irradiation.^[^ ^90^ ^]^ For comparison, pristine MPG shows a reflectance (≈2.5%), while pure graphene exhibits near‐zero reflectance (< 0.2%). These results suggest that the MPG component within the composite structure contributes to heat retention, likely by suppressing radiative losses.

To assess the photothermal behaviour, the temperature progression during 60 min under one sun irradiation was recorded using an IR camera, followed by natural cooling. As shown in Figure 3c, the surface temperature increased within five minutes. Before irradiation, the surface temperature of LM–Graphene–MPG, Graphene–MPG, and MPG were 26.4, 26.3, and 26.2 °C, respectively. After 60 min of exposure, the surface temperature of the gels reached 54.7, 53.7, and 55.5 °C, respectively, which is consistent with their broadband light absorption enabling efficient photothermal heating. In wet conditions (Figure 3d), the gel efficiently transferred heat to the water, leading to evaporation. Consequently, the maximum temperature of the wet gel (LM–Graphene–MPG) reached only 41.22 °C, which is less than half what we measured for the dry gel. This difference is largely due to the endothermic nature of the evaporation process, which absorbs heat and lowers the overall temperature. Subsequently, the photothermal performance of the samples under visible light was evaluated under 532 nm laser irradiation (Figure S13, Supporting Information). The surface temperature of the LM–Graphene–MPG reached 54 °C, which was significantly higher compared to MPG (41 °C) and graphene–MPG (37 °C). The NIR photothermal performance was then assessed using 830 nm NIR laser irradiation, LM–Graphene–MPG showed a rapid temperature increase, reaching ≈119 °C, followed by MPG at 85 °C and Graphene–MPG at 69 °C (Figure S13, Supporting Information). Also, under 1064 nm laser irradiation (Figure S13, Supporting Information), under this condition, LM–Graphene–MPG showed a rapid temperature increase, reaching ≈129 °C, followed by MPG at 97 °C, and Graphene–MPG at 60 °C. At 830 and 1064 nm, MPG clearly dominates, as it outperforms graphene–MPG significantly. The LM–Graphene–MPG composite achieves the highest temperatures under all laser conditions, especially NIR range (up to 129 °C), demonstrating a clear synergistic effect.

The enhanced light absorption and photothermal performance of LM–Graphene–MPG result from the combined contributions of its components. Graphene primarily contributes through broadband light absorption^[^ ^54^ ^]^ in the UV‐VIS and lower NIR regions, enabled by its zero‐bandgap structure and strong π–π interactions, with and superior thermal conductivity^[^ ^55^ ^]^ facilitates efficient heat distribution.^[^ ^91^ ^]^ On the other hand, the gel network (MPG) composed of TA–Fe^3+^–Ti^4+^ complexes– serves as the dominant absorber in UV (due to aromatic rings in TA), visible, and NIR region due to LMCT bands. Additionally, the gel matrix contributes in reducing conductive heat losses, achieving temperatures of 85–97 °C under 830–1064 nm lasers. Furthermore, its resistance to salt contamination and ability to confine thermal energy at the interface promote rapid vapor generation. Although LM component does not contribute significantly to light adsorption, it enhances overall thermal management due to its high thermal conductivity and scattering effects.^[^ ^92^ ^]^ Overall, the metal‐phenolic gel provides broadband light absorption and hydrophilicity for efficient water transport, graphene nanosheets contribute to enhancing the light absorption and rapid photothermal conversion, and liquid Ga particles contribute high thermal conductivity with localized heat confinement to minimize energy loss; together, these complementary functions (Figure S13, Supporting Information) create a hierarchical composite that synergistically enhances solar‐to‐thermal conversion efficiency.

To evaluate the light‐to‐heat conversion ability of our materials, we determined the photothermal conversion efficiency (η) based on an energy balance model, where the heat transfer coefficient (h) was extracted from the cooling curve analysis (Note S1 and Figure S14, Supporting Information). Using this method, the LM–Graphene–MPG sample exhibited high efficiencies of 99% at 532 nm, 75% at 830 nm, and 67% at 1064 nm (Table S1). These values demonstrate the strong broadband photothermal response of the composite across the visible and near‐infrared regions, with particularly efficient conversion under green (532 nm) excitation.

Thermal conductivity measurements (Figure 3e) reveal that LM–Graphene–MPG has a conductivity of 0.37 W m^−1^ K^−1^ slightly above the 0.32 W m^−1^ K^−1^ conductivity of MPG, but still notably lower than water (0.592 W m^−1^ K^−1^). This low thermal conductivity helps concentrate heat at the photothermal interface by minimizing losses, a feature typically achieved in gels through trapping air pockets (air has a conductivity of ≈0.024 W m^−1^ K^−1^) and leveraging a high specific surface area.^[^ ^93^ ^]^ With the lowest thermal conductivity, it can block the heat diffuse down to the bulk water significantly.^[^ ^94^ ^]^ By limiting conduction, convection, and radiation pathways, the MPG–based gels effectively localize heat where it is most needed. Meanwhile, the moderate specific heat capacity of LM–Graphene–MPG means less energy is required to raise its temperature, further enhancing efficiency. Notably, the measured thermal conductivity aligns well with other reported evaporator hydrogel (≈0.27 W m^−1^ K^−1^),^[^ ^95^ ^]^ coconut husk TA–Fe^3+^ (≈0.18 W m^−1^ K^−1^),^[^ ^96^ ^]^ PVA/poly(acrylamide) without ethylene glycol/Cellulose nanocrystal‐EGaIn/PANI hydrogel (≈0.3978 W m^−1^ K^−1^),^[^ ^97^ ^]^ Le/CNTs‐corn stalk (≈ 0.125 WmK^−1^),^[^ ^98^ ^]^ and sponge‐TA@APTES@Fe^3+^ (≈0.23 W m^−1^ K^−1^)^[^ ^46^ ^]^ supporting its suitability for efficient solar steam generation. The LM particles mixture with Graphene creates a thermally conductive network.

In addition to its photothermal properties, LM–Graphene–MPG, graphene–MPG composites exhibit high electrical conductivity, reaching 1118 and 83 784 S m^−1^. This was sufficient to power an LED lamp when integrated into a simple electrical circuit (Figure S15 and Table S2, Supporting Information). To evaluate electricity generation during seawater evaporation, LM–Graphene–MPG was connected to two gold electrodes. The possible ion migration generated a stable potential difference via electrical double layer and streaming effects,^[^ ^98^, ^99^ ^]^ delivering an open circuit voltage of 162 mV and a short circuit current of 69 nA under one sun (Figure S15, Supporting Information).

### 2.3 Water Evaporation Performance

**Figure 4:**
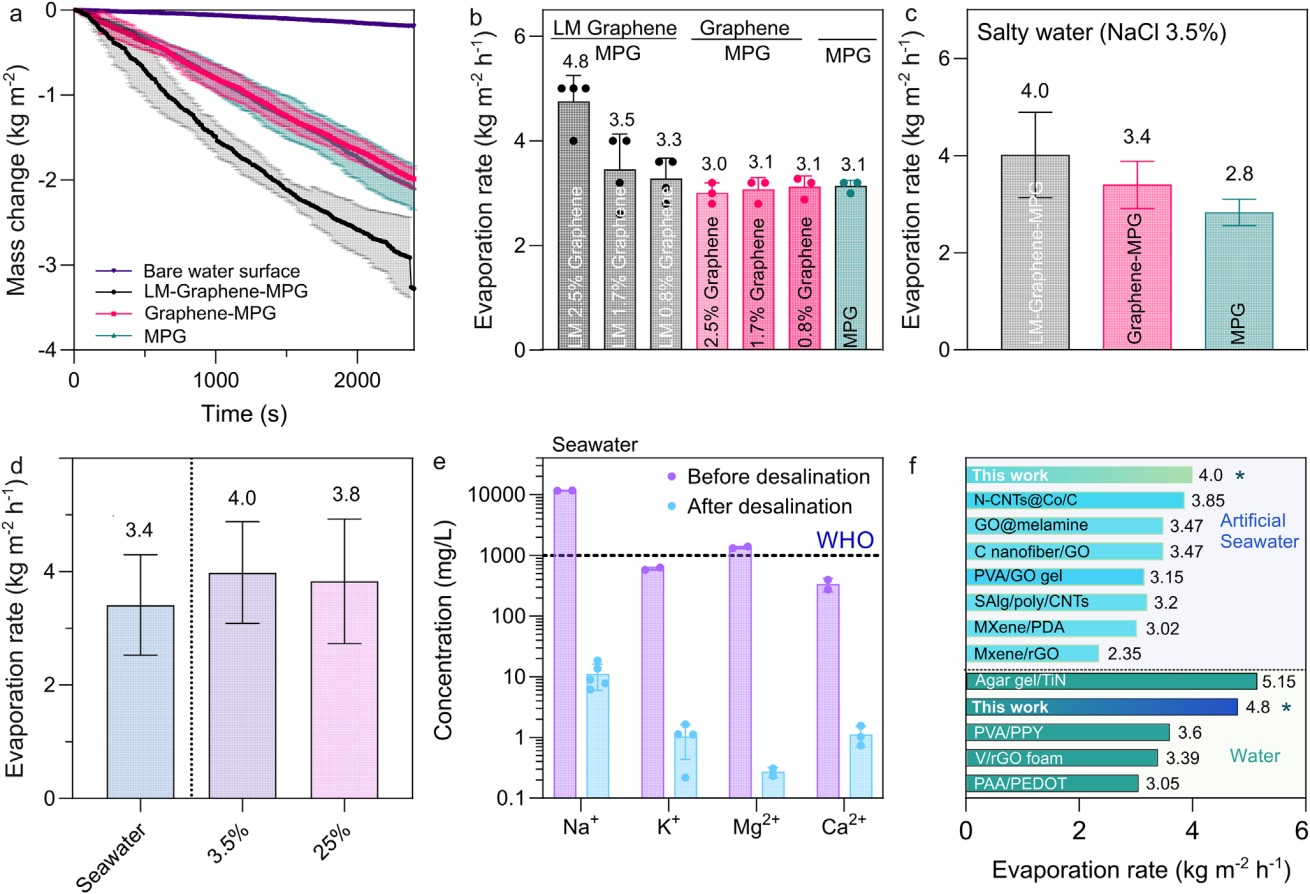
a) Water evaporation over time under one sun illumination. b) Evaporation rate (DI water) under one sun illumination. c) Evaporation rate of saline water (3.5% NaCl) under one sun illumination. d) Evaporation rate of different concentrations of NaCl under one sun illumination. e) Main ion concentrations (Na^+^, Mg^2+^, K^+^, Ca^+^) in the original seawater and the collected clean water from solar evaporation, measured by inductively coupled plasma optical emission spectroscopy (ICP‐OES). f) Comparison of evaporation rate to similar systems reported in the literature. Details of the materials and their acronyms used in these studies can be found in the Tables S3 and S4 (Supporting Information).

The evaporation rate of the interfacial evaporator equipped with different gel samples was measured. The evaporator placed over deionized (MilliQ) water was exposed to one sun illumination, and the real‐time mass change under continuous illumination was recorded (**Figure** **4**a). The water evaporation rate on the surface of bare water (without photothermal materials) was measured to be 0.046 and 0.3 kg m^−2^ h^−1^ under dark and one sun illumination (solar irradiance of 1 kW m^−2^), respectively. We then examined how varying graphene concentrations (2.5,1.7, and 0.8%) influenced the evaporation performance (Figure 4b). While adding graphene alone led to similar evaporation rates, combining graphene with Ga substantially improved the gel's overall evaporation rate, with LM–Graphene–MPG reaching 4.8 kg m^−2^ h^−1^ under one sun. In contrast, Graphene–MPG and MPG exhibited lower rates, at 3.0 and 3.1 kg m^−2^ h^−1^, respectively. LM–Graphene–MPG represents an evaporation increase of (60%) as compared to the Graphene‐MPG and MPG.

To investigate the effect of absorber shape on evaporation performance, the LM–Graphene–MPG was moulded into cylindrical (2D) and conical (3D) shapes with the same footprint area. As shown in Figure 1g, the conical (3D) shape reached an evaporation rate for DI water (MilliQ) of 4.8 kg m^−2^ h^−1^ after one hour of simulated solar irradiation, surpassing that of the cylindrical design with an evaporation rate of 2.52 kg m^−2^ h^−1^. This improvement can be attributed to the larger evaporative surface area,^[^ ^100^ ^]^ which leads to higher local temperatures (44.4 °C at the centre and 37.8 °C at the periphery), in contrast to the 2D interface ≈38 °C at the centre (Figure 3f). Suggesting the system's reduced heat exchange with the environment, enhanced solar capture, and larger evaporation area. We observed that the sidewall temperature was 4 °C lower than the illuminated top surface (IR thermal imaging, Figure S16, Supporting Information). In addition, the 3D evaporators utilize side surfaces kept relatively cooler than the environment, enabling additional energy harvesting through radiation and convection (Note S2, Supporting Information).^[^ ^101^, ^102^ ^]^ The sidewalls that are not exposed to direct sunlight drop below the ambient temperature due to evaporative cooling effects, leading to heat absorption from the environment to sustain further evaporation.^[^ ^103^, ^104^ ^]^

Next, we explored the gel evaporator's desalination capabilities, where the evaporation rate was tested in water with 3.5% of NaCl (Figure 4c). These results were comparable to those obtained with MilliQ water, where the LM–Graphene–MPG exhibited the highest evaporation rate with 4.0 kg m^−2^ h^−1^, followed by MPG with 2.8 kg m^−2^ h^−1^. Additionally, the evaporation performance of LM–Graphene–MPG was evaluated with various NaCl concentrations (3.5 and 25%) and, as well in seawater. As shown in Figure 4d, the steam generation of the gel evaporator declined as salt concentration increased. The reduced evaporation rates and efficiencies 25 wt.% NaCl solutions could be ascribed to the lower water vapor pressure under high salinity conditions. With a 25 wt.% NaCl solution, the LM–Graphene–MPG showed an evaporation of 3.8 kg m^−2^ h^−1^. Notably, no salt precipitation was observed on the surface of the gel during the evaporation, indicating excellent salt tolerance. Furthermore, 0.5 g of solid NaCl was put on the upper surface of the MPG sample, which was then floated on simulated seawater (3.5% NaCl). And the above amount of salt dissolved after 277 min in simulated seawater (Figure S17, Supporting Information). The pores of MPG can serve as a continuous water transport through which salt can move from the upper evaporation layer (higher salinity) to the bulk simulated seawater (lower salinity). The driving for salt movement is a diffusion effect caused by the salinity difference during salt rejection. Therefore, salt crystallization on the MPG surface could be prevented.

We further evaluated the performance of the composite using a real seawater sample. The evaporation rate for seawater was found to be 3.4 kg m^−2^ h^−1^ (Figure S18 and S19, Supporting Information). To assess the quality of the evaporated water, the concentrations of four representative ions were measured in both seawater and the collected vapor condensate. As shown in Figure 4e, the concentration of ions decreased, and the concentrations of Na^+^, Mg^2+^, K^+^, and Ca^2+^ ions were 11.0, 0.3, 1.0, 1.1 mg L^−1^ after desalination respectively. These values comply with World Health Organization (WHO) standards for safe drinking water.^[^ ^105^ ^]^

To explore the potential of our photothermal composite for wastewater treatment, we also assessed its ability to remove organic contaminants using methylene blue as a model pollutant. As shown in the photographs (Figure S20 and S21, Supporting Information), the initially blue solution turned clear after evaporation and condensation, indicating the effective pollutant removal. This result was further supported by UV–vis spectroscopy, where the characteristic absorption peak of methylene blue at 662 nm disappeared, confirming high removal efficiency.

Since the evaporation rate reflects the prospect of solar steam generation, the performance of the developed gel evaporator was compared with other published work; the results are shown in Figure 4f (Table S3, Supporting Information), including Mxene/rGO,^[^ ^106^ ^]^ Mxene/PDA,^[^ ^107^ ^]^ Salg/poly/CNTs,^[^ ^108^ ^]^ Ca nanofiber/GO,^[^ ^109^ ^]^ and GO@melamine.^[^ ^110^ ^]^ The superior evaporation performance of the developed gel could be explained as follows. On one hand, the broadband absorption and the efficient solar thermal conversion resulted in dramatic heat generation. On the other hand, the hydrophilic gel network facilitated effective water transport and the poor thermal conductivity of the evaporator. The results indicate its potential application in practical solar vapor generation. These underscore the material's potential for practical solar vapor generation applications.

For practical application, an outdoor desalination experiment was carried out in Sydney, Australia, using a transparent PMMA condenser under natural sunlight. The test was conducted from 10:25 AM to 5:25 PM on a cloudy day, with ambient temperature, solar intensity, UV index, wind speed, and humidity recorded every 15 min (Figure S22, Supporting Information). Over 6 h of irradiation, the prototype produced 3.35 kg m^−2^ of purified water under an average solar flux of 171.1 W m^−2^ and an ambient temperature of 24.7 °C (Figure S23, Supporting Information). Based on laboratory‐scale fabrication, the estimated cost of our evaporator system (area 6.249 cm^2^) was ≈$0.24/cm^2^ (Table S5 and S6).

## 3 Conclusion

This study presents a novel photothermal composite for ISSG by combining graphene, liquid metal Ga particles, and a MPG. These components work synergistic to enable efficient broadband light absorption, heat localization, and water transport— key challenges in ISSG. Graphene and the MPG matrix provide broad‐spectrum solar absorption, while Ga‐based LM particles boost NIR utilization and heat localization. In addition, the MPG network ensures structural integrity, hydrophilicity, and thermal retention. Under simulated solar irradiation, the LM–Graphene–MPG composite delivers high evaporation rates, excellent salt rejection, and sustained stability, demonstrating its promise for scalable, sustainable seawater desalination. These findings contribute to the advancement of the next‐generation solar‐driven desalination technologies and offer a compelling solution to global water scarcity.

## 4 Experimental Section

### Materials

Gallium (Ga, beads, 99%) was purchased from Roto Metals, USA. Tannic acid, iron (III) nitrate nonahydrate (Fe(NO_3_)_3_·9H_2_O), graphene nanoplatelets 5 µm particle size, and titanium (IV) bis(ammonium lactato) dihydroxide solution (50 wt.% in H_2_O; Ti‐BALDH) were obtained from Sigma–Aldrich. 2‐Propanol AR grade was obtained from Chem‐supply. Dimethyl sulfoxide (DMSO) was purchased from AJAX Finechem. High‐purity (Milli‐Q) water with a resistivity of 18.2 MΩ cm was obtained from an inline Millipore RiOs/Origin water purification system.

### Preparation of LM–Graphene–Metal Phenolic Gel

Here, Ga was first melted using a water bath maintained at 60 °C. An aliquot of the molten Ga (100 µL) was then added to a (50 mL) centrifuge tube containing a graphene nanoplatelet suspension (7.5 mL, 200 mg mL^−1^ in isopropanol). The resulting dispersion was sonicated for 20 min using a probe sonicator (VCX 750, Sonics & Materials, Inc., tip no 630‐0420‐B) at 50% amplitude with a pulsing sequence (6s on, 4s off). To prevent sample heating during sonication, the tube was placed in an ice‐water bath.

Next, a TA solution (5.2 mL of 25% w/w, dissolved in isopropanol: water [64:36% w/w]) was added, and the mixture was sonicated under the same conditions for another 20 min. Following this, Fe(NO_3_)_3_·9H_2_O (2.5 mL of 200 mg mL^−1^ in water) was added under vortex mixing for ≈40 s. Finally, Ti‐BALDH: DMSO solution (1.5 mL, 50:50% v/v) was introduced under vortex mixing for another ≈40 s to form the LM–Graphene–MPG composite. The resulting gel was left to dry under a fume hood for 36 h.

### Characterizations and Methods

In this work, SEM images were obtained using (Zeiss Sigma VP HD, Carl Zeiss Microscopy, WP, United States) after gold coating. EDS mapping was obtained using at an accelerating voltage of 20 kV. Size distributions of the particles were determined with a Malvern Zetasizer Nano‐ZS by the Dynamic Light Scattering technique. Raman spectroscopy was performed using a Renishaw InVia Qontor microscope (Renishaw plc., Wotton‐under‐Edge, UK) equipped with an air‐cooled charge‐coupled device (CCD) detector. The system included holographic notch filters and a 2400 lines/mm grating (for visible and UV regions). A Leica DMLM microscope fitted with a standard 50× objective, which was used for sample focusing. The spectrometer was operated via Renishaw WiRE software (Version 5.6). Instrument calibration was carried out prior to measurement using a silicon internal standard. Raman excitation was provided by a Renishaw RL532C continuous‐wave diode laser (λ = 532 nm). Spectra were acquired using the 50× objective across a spectral range of 70–3200 cm^−1^. To analyze the crystallographic structures of the samples, each powder was loaded into a 0.5 mm deep cavity of a glass sample holder. Powder XRD data were collected in Bragg–Brentano geometry using a Rigaku SmartLab SE powder diffractometer equipped with Cu Kα radiation (Kα_1_ = 1.540598 Å, Kα_2_ = 1.544426 Å), operating at 40 kV and 40 mA. The setup included a 10 mm length‐limiting slit and ½° divergence slits. Diffraction patterns were acquired from spinning samples using an XSPA‐400 ER 2D detector, with continuous scanning at 5°/min over a 2θ range of 5–90°. XPS was performed using a K‐Alpha+ spectrometer (Thermo Scientific) equipped with an Al Kα source and a 180° double‐focusing hemispherical analyzer with a 128‐channel detector. All spectra were acquired with the flood gun activated. Data processing was carried out using Thermo Advantage software, with energy calibration referenced to the C 1s peak at 284.8 eV. UV–Vis absorption spectra were recorded using a UV‐3600 Plus spectrophotometer (Shimadzu, Japan) equipped with an integrating sphere. Absorbance values were calculated using the equation: A = 100 – %R, where *R* denotes the reflectance. Reflectance spectra in the IR range of 1.6–25 µm were measured using a Fourier Transform Infrared (FTIR) spectrometer (Alpha, Bruker Optiks GmbH, Germany) equipped with a QuickSnap External Reflection Module and a DTGS detector. The standard sample spot size of 5 mm was reduced to 3 mm using a sample mask, and 64 scans were acquired per measurement. A background spectrum was recorded using a magnetically mounted gold‐coated reference mirror under the same acquisition conditions as the sample. Thermal conductivity at room temperature was evaluated using a Thermtest TPS RT system with a sensor diameter of 12.8 mm, connected to the Thermtest Measurement Platform‐1 (MP1). Contact angle was measured by an Optical Tensiometer (Theta Flex Auto 2, Biolin Scientific).

The ion concentration of the seawater and desalination water was measured with inductively coupled plasma optical emission spectroscopy (ICP‐OES) (Avio 500, Perkin Elmer). To characterize the photothermal properties of LM‐graphene‐MPG, the samples were put in a glass slide and irradiated by 830 nm laser (spot size: ≈8.4 µm, Renishaw, objective x5, laser power 72.23 mW) for 15 min. An optical power meter (PM100USB, Thorlabs) was used for laser power measurements. The surface temperatures were recorded using an infrared camera (FLIR ONE Edge Pro, Teledyne FLIR LLC).

### Study of Thermal Degradation using Evolved Gas Analysis

Evolved Gas analysis (TGA–FTIR) was used to monitor the degradation products of LM‐Graphene‐MPG. In the experiment, TGA equipped with an appropriate gas purge was connected with a TGA–FTIR interface (TGA‐IR and TRG 004) installed inside the infrared spectrometer (Bruker Invenio FTIR). Nitrogen was typically purged at 50 mL min^−1^. About 15 mg of the sample was used in each test. The thermal degradation was studied under nitrogen and air, respectively, at a heating rate of 10 °C min^−1^ from room temperature to 500 °C. The temperature of the transfer pipe was set at 200 °C while the TGA–FTIR sample cell was set at 200 °C to prevent the condensation of volatiles in the sample chamber. The data from the infrared spectrometer comprises a series of IR spectra measured at an average of 32 scans with 4 cm^−1^ resolution in the range of 4000 to 650 cm^−1^. The IR spectra were compared with standard spectra of organic species reported in comparison with standard gas phase FTIR spectra from NIST Chemistry WebBook and vapor phase library EPA‐NIST.

### Solar Steam Generation Tests

Performance measurements were conducted using a solar simulator (SunLite 11002‐2, Abet Technologies, USA, ASTM Class A) equipped with a 100 W Xe arc lamp as the light source. The light power density was verified using a solar power meter (QM 1582, Digitech). Surface temperature of the composite samples was monitored using an infrared camera (P2 prop, Infiray), with an accuracy of ± 2 °C. The water mass loss was determined using an electronic balance (BCE224I‐1S, Sartorius). All experiments were performed at a controlled ambient temperature of 20 ± 2 °C.

The water evaporation rate of each sample was calculated using the following equation:

(1) $$m=\frac{\Delta m}{S\;\times \;t}$$

where *m* is the water evaporation rate (kg m^−2^ h^−1^), Δ*m* is the mass change of water in 1 h (kg), and *S* is the projected area of the top surface (m^2^), the side and outer wall area was not included in S, *t* is the irradiation time (h).

### Outdoor Experiment

A Pantech weather station model GW1201was used for measuring ambient temperature, solar intensity, UV index, wind speed, and humidity, recorded every 15 min.

### Statistical Analysis

GraphPad Prism v10 was generally used for the mathematical and statistical presentation of the data. Data is presented as mean ± standard deviation.

## Conflict of Interest

The authors declare no conflict of interest.

## Author Contributions

N.F. and M.A.R. conceived the idea and designed the experiments. N.F. conducted the experiments, performed physical characterizations with the help of F.C., H.B., Y.W., S.C., M.S.W., N.A., L.L., M.H.K. The following authors contributed to the data analyses, scientific discussions, and preparation of the manuscript: M.B.G., F.‐M.A., M.A.R., F.C., S.S. and K.K.‐Z. All authors revised the manuscript and provided valuable comments.

## Supporting information



Supporting Information

## Data Availability

The data that support the findings of this study are available in the supplementary material of this article.
