# Supplementary material for: Liquid Metal–Polyphenol Hybrids for Solar Steam Generation
Source: Adv Sci (Weinh). 2025 Oct 13;12(48):e12789. doi: 10.1002/advs.202512789 (PMC12752657; doi:10.1002/advs.202512789)
Supplement: Supplementary file 1 — Supporting Information [file ADVS-12-e12789-s001.pdf]

Supporting Information

Liquid Metal–Polyphenol Hybrids for Solar Steam Generation

Nieves Flores, Franco Centurion, Md. Hasan Al Banna, Nur-Adania Nor-Azman, Moonika. S. Widjajana, Yuqin Wang, Li Liu, Shih-Hao Chiu, Majharul Haque Khan, Sarina Sarina, Mohammad B. Ghasemian, Francois-Marie Allioux, Kourosh Kalantar-Zadeh, Md. Arifur Rahim*.*

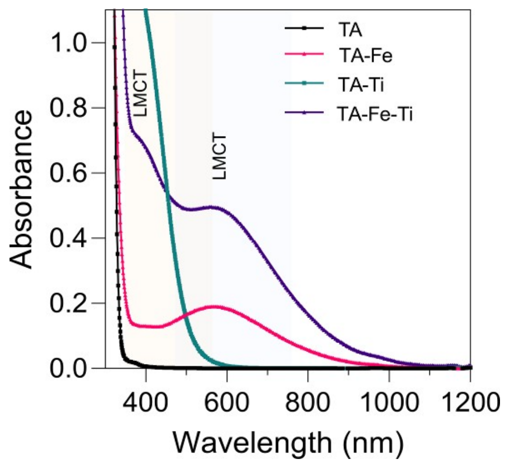

Figure S1. UV–vis absorption spectra of MPG (TA–Fe–Ti), TA–Fe^{3+}, TA–Ti^{4+}, and pure TA.

UV-vis spectroscopy measurements were conducted to analyse TA–Fe^{3+} ligand-metal charge transfer bands (LMCT, 440 – 700 nm wavelength) and π-π* transition (~300 nm wavelength). The ligand-metal charge transfer LMCT peak can describe the coordination complex state of metal-phenolic networks (mono-complex: ~700 nm, bis-complex: ~570 nm, and tris-complex: ~460 nm),[1] and the π - π * transition peak was observed at 300 nm originating from the aromatic ring of TA. For the MPG sample, and TA–Fe^{3+} sample, the LMCT peak position was 572 nm indicating TA–Fe^{3+} to be dominant in bis-complex mode.

The ligand-metal charge transfer for TA–Ti^{4+} : LMCT peak can describe the coordination complex state of metal-phenolic networks (bis-complex ~ 350 nm[2], tris-complex: ~378 nm). The LMCT peak position was 374 nm indicating the TA–Ti^{4+} tris-complex.

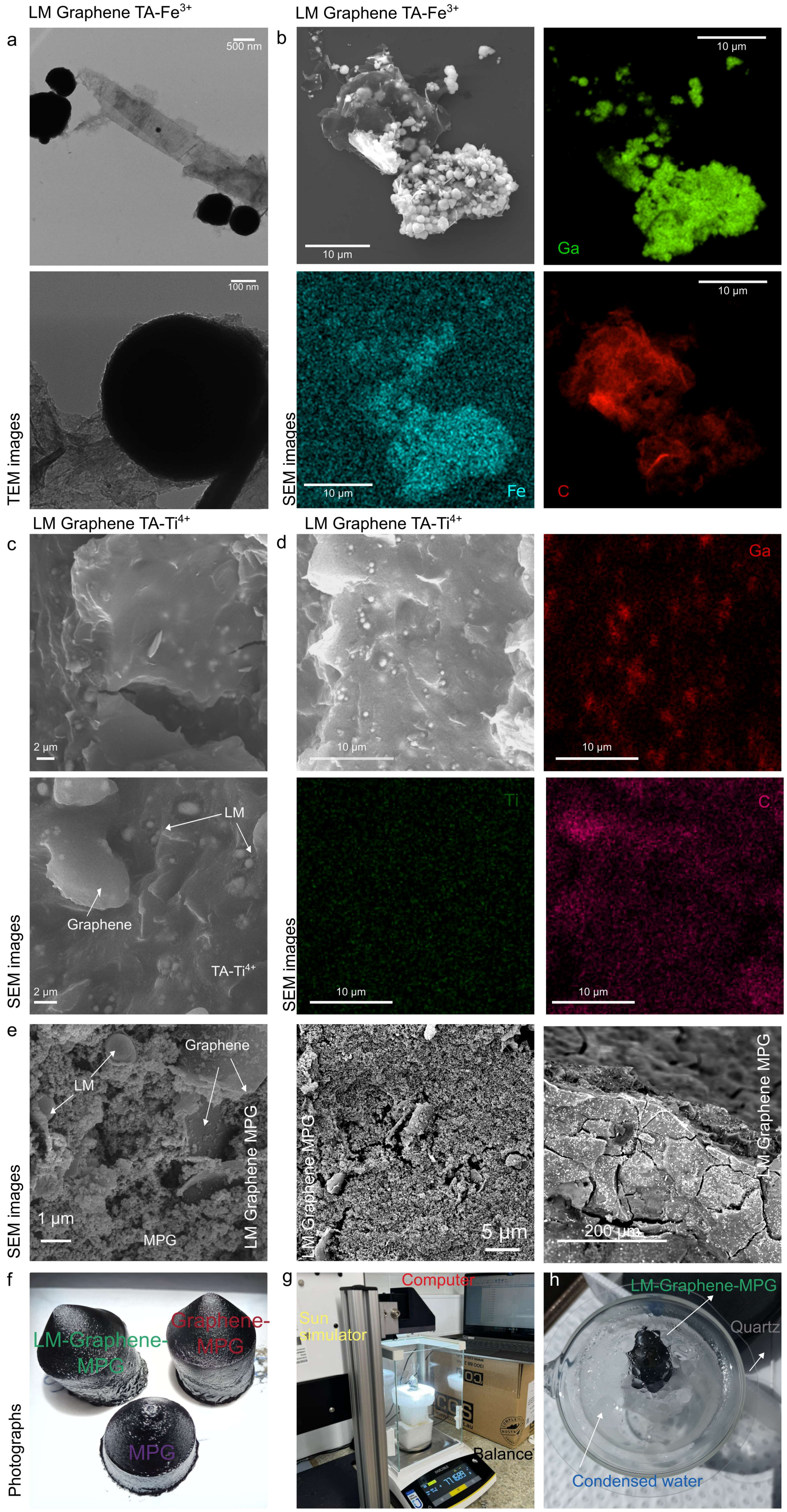
LM Graphene TA-Fe3+
a
500 nm
100 nm
TEM images
LM Graphene TA-Fe3+
b
10 µm
Ga
Fe
C
SEM images
LM Graphene TA-Ti4+
c
2 µm
LM
Graphene
TA-Ti4+
SEM images
LM Graphene TA-Ti4+
d
10 µm
Ga
Ti
C
SEM images
e
Graphene
LM
1 µm
MPG
LM Graphene MPG
5 µm
200 µm
SEM images
f
LM-Graphene-MPG
Graphene-MPG
MPG
Photographs
g
Computer
Sun simulator
Balance
h
LM-Graphene-MPG
Quartz
Condensed water

Figure S2. a) TEM images of Ga particles on graphene, b) SEM images of LM–Graphene–TA–Fe^{3+}. Element mapping: Ga, Fe, C. c) SEM images of LM–Graphene–TA–Ti^{4+}. d) SEM images of LM–Graphene–TA–Ti^{4+}. Element mapping: Ga, Ti, C. e) SEM images of LM–Graphene–MPG, f) Photographs of LM–Graphene–MPG, Graphene–MPG, and MPG samples, g) Photographs of device for evaluation of solar-powered steam performance, h) Photographs of the solar evaporator generating steam.

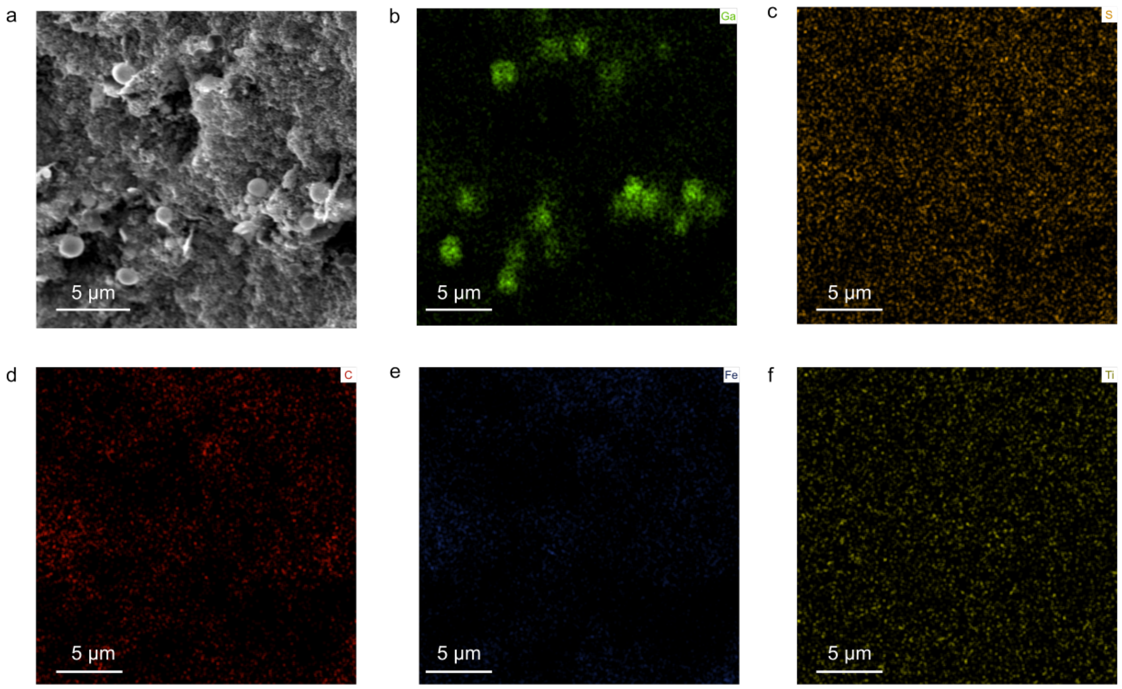

Figure S3. SEM images of LM–Graphene–MPG with corresponding element mapping: b-f) Ga, S, C, Fe, Ti.

Note that the S signal possibly originated from some residual DMSO (used during MPG synthesis) coordinated with Ti^{4+} centres in the MPG network (Figure S3–S7).

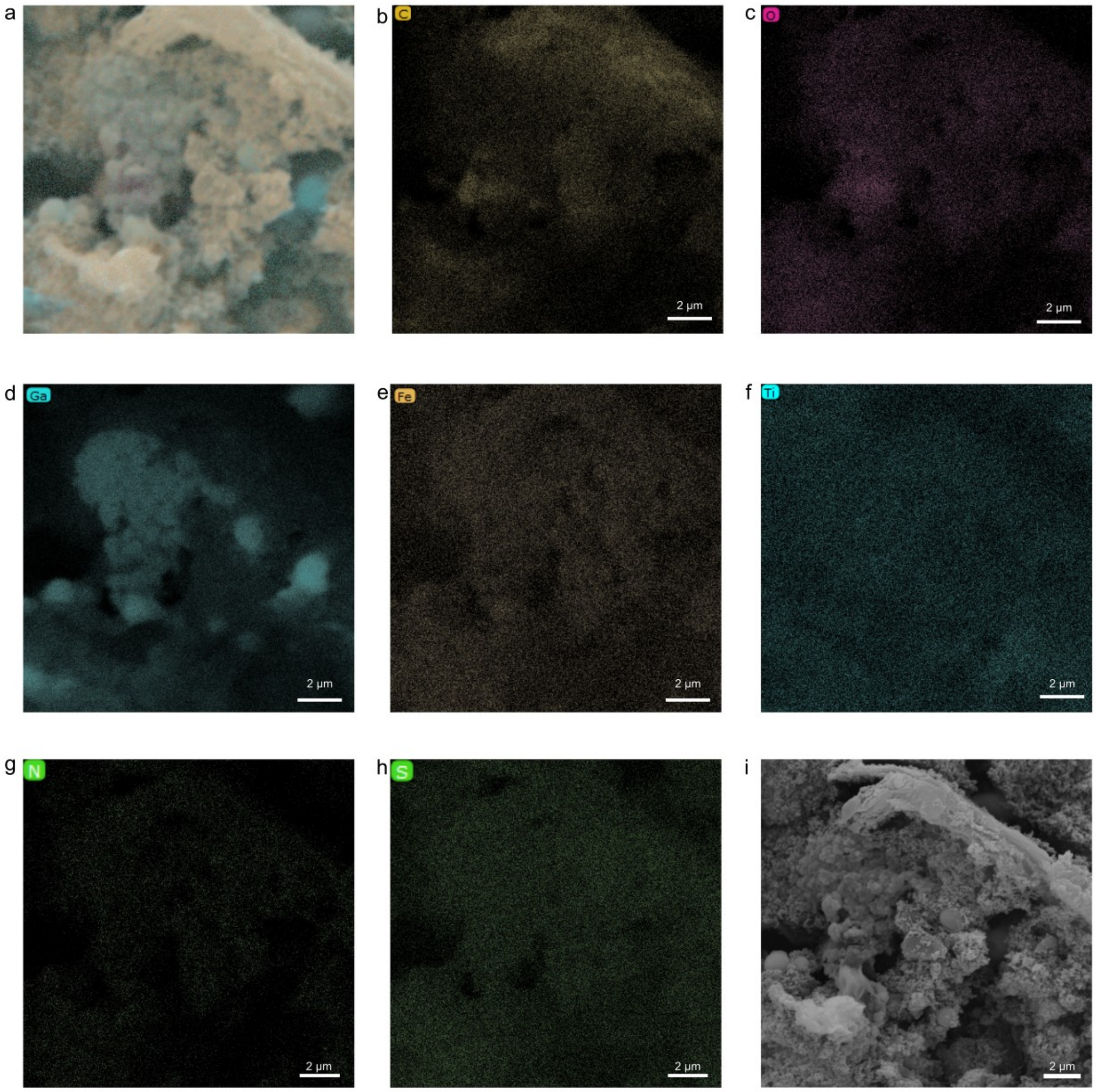

Figure S4.SEM image of LM–Graphene–MPG with corresponding element mapping: a) overlap, b-h) C, O, Ga, Fe, Ti, N, S. i) electron.

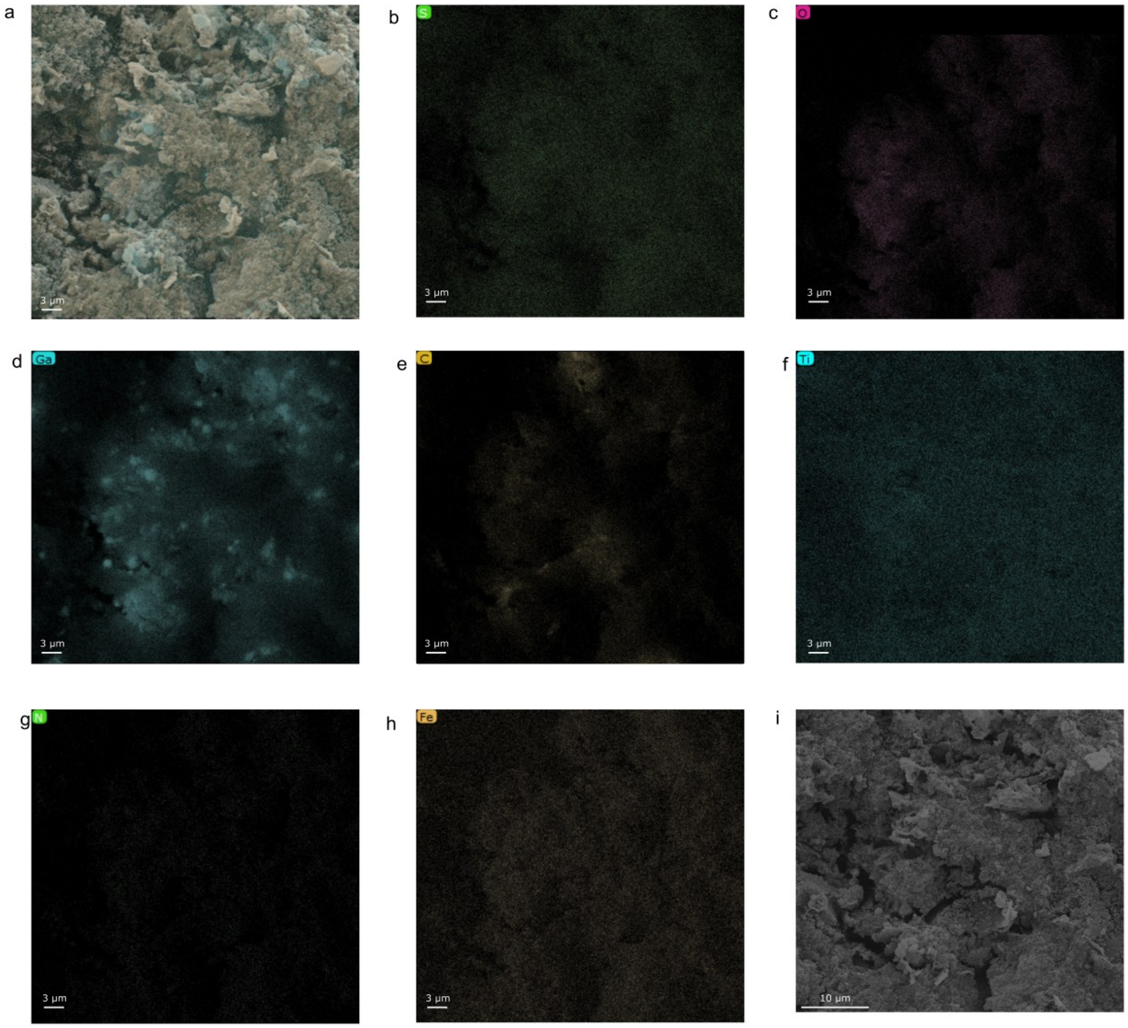

Figure S5. SEM of LM–Graphene–MPG with element mapping a) overlap, b-h) S, O, Ga, C, Ti, N, Fe. i) electron.

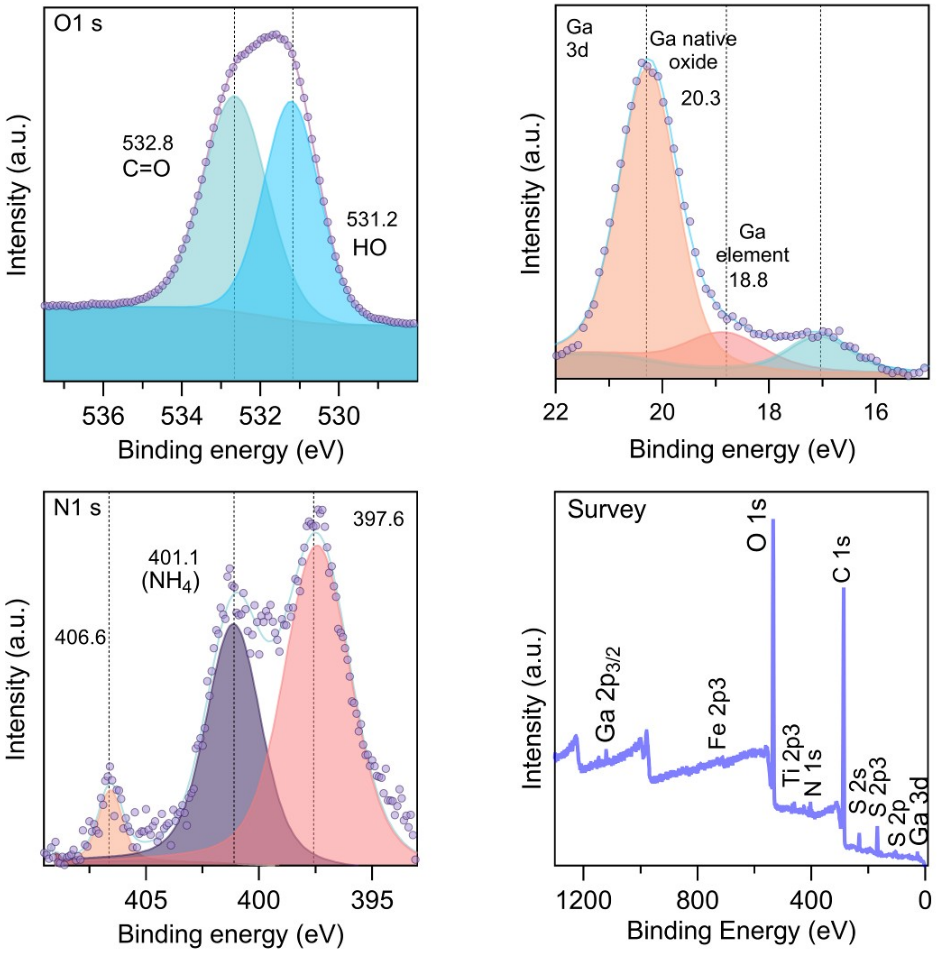

Figure S6. The XPS spectra of the LM–Graphene–MPG. (a) O 1s core level spectra, (b) Ga 3d spectra (c) N 1s core level spectra, (d) Survey spectra showing the presence of Ga 2p, Fe 2p3, O 1s, Ti 2p3, N 1s, C 1s, S 2s, S2p3, and Ga 3d.

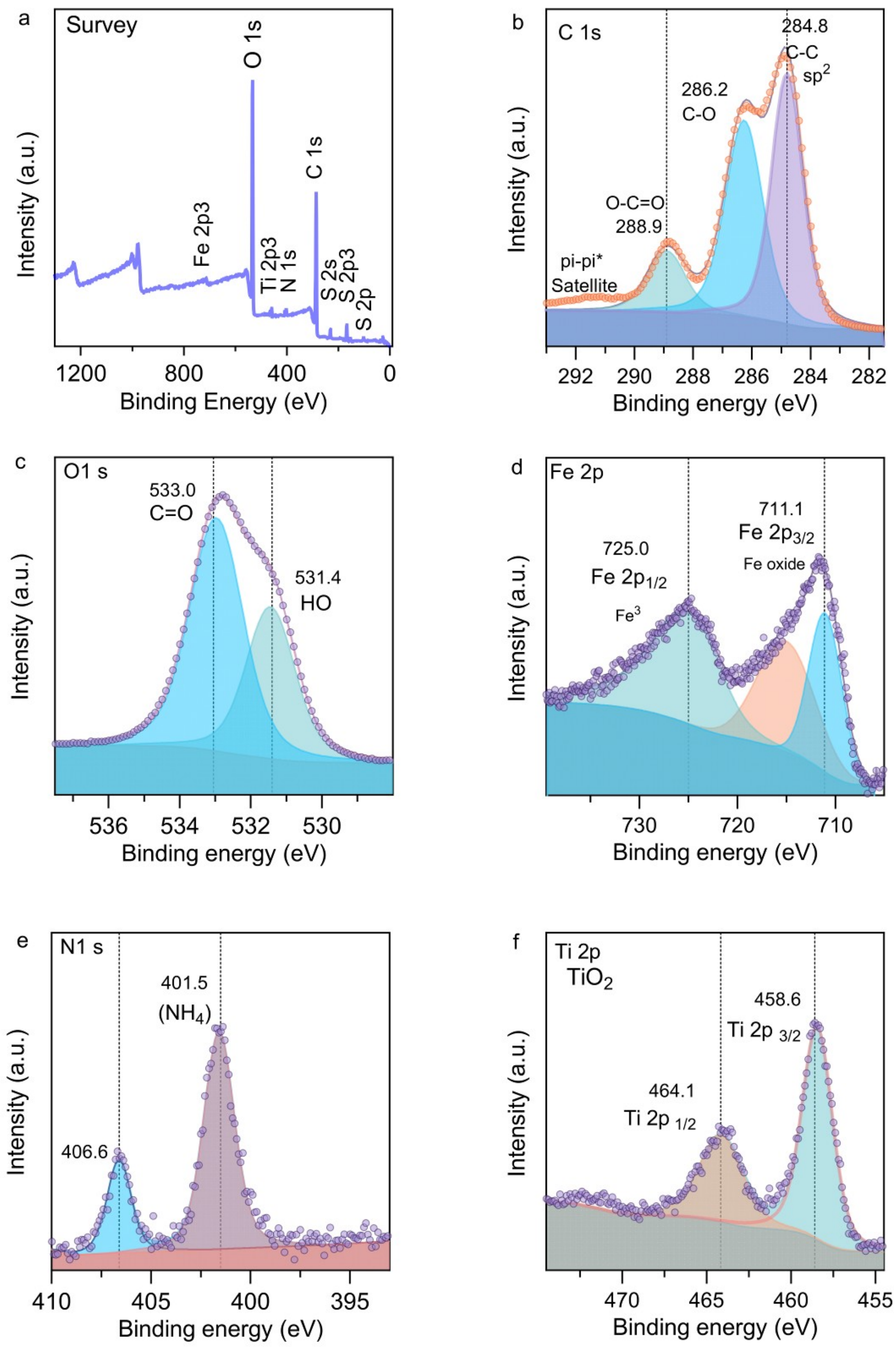

Figure S7. The XPS spectra of the MPG. (a) Survey spectra showing the presence of Fe 2p3, O 1s, Ti 2p3, N 1s, C 1s, S 2s, and S2p3, (b) C 1s spectra (c) O 1s spectra, (d) Fe 2p spectra, (e) N 1s spectra, (f) Ti 2p spectra.

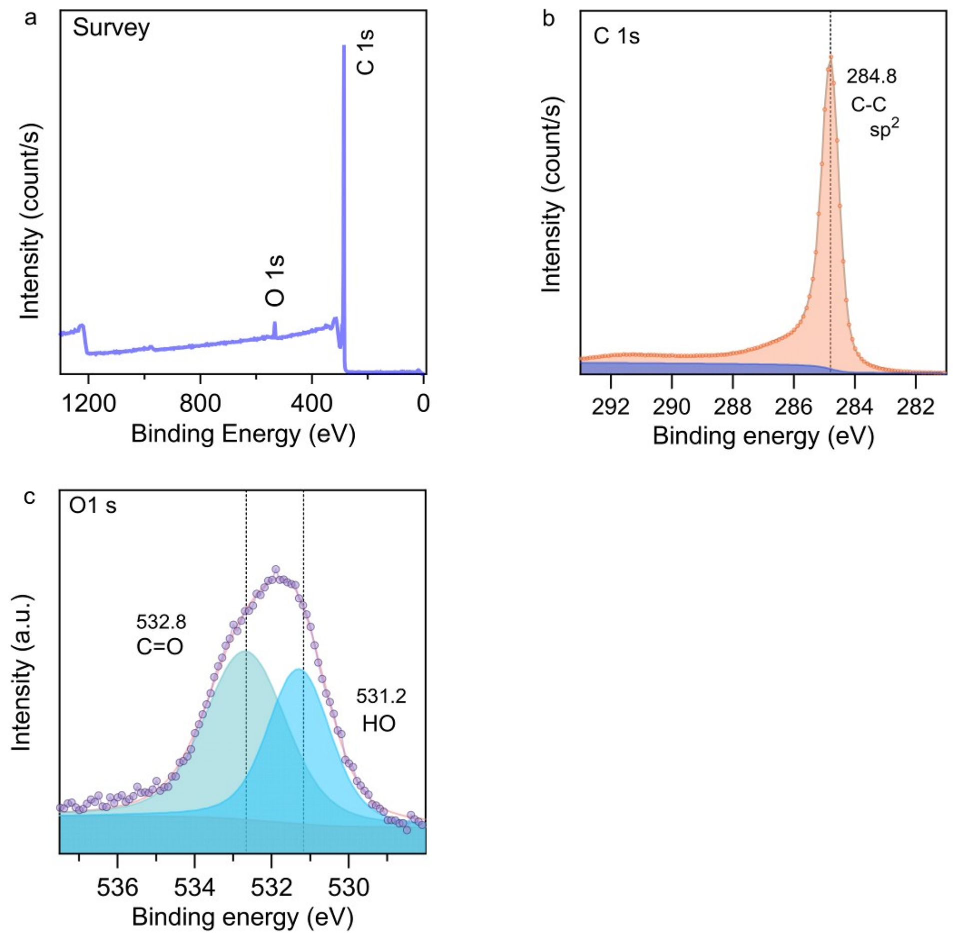

Figure S8. The XPS spectra of the graphene nanoplatelets. (a) Survey spectra showing the presence of O 1s, C 1s, (b) C 1s core level spectra, (c) O 1s core level spectra.

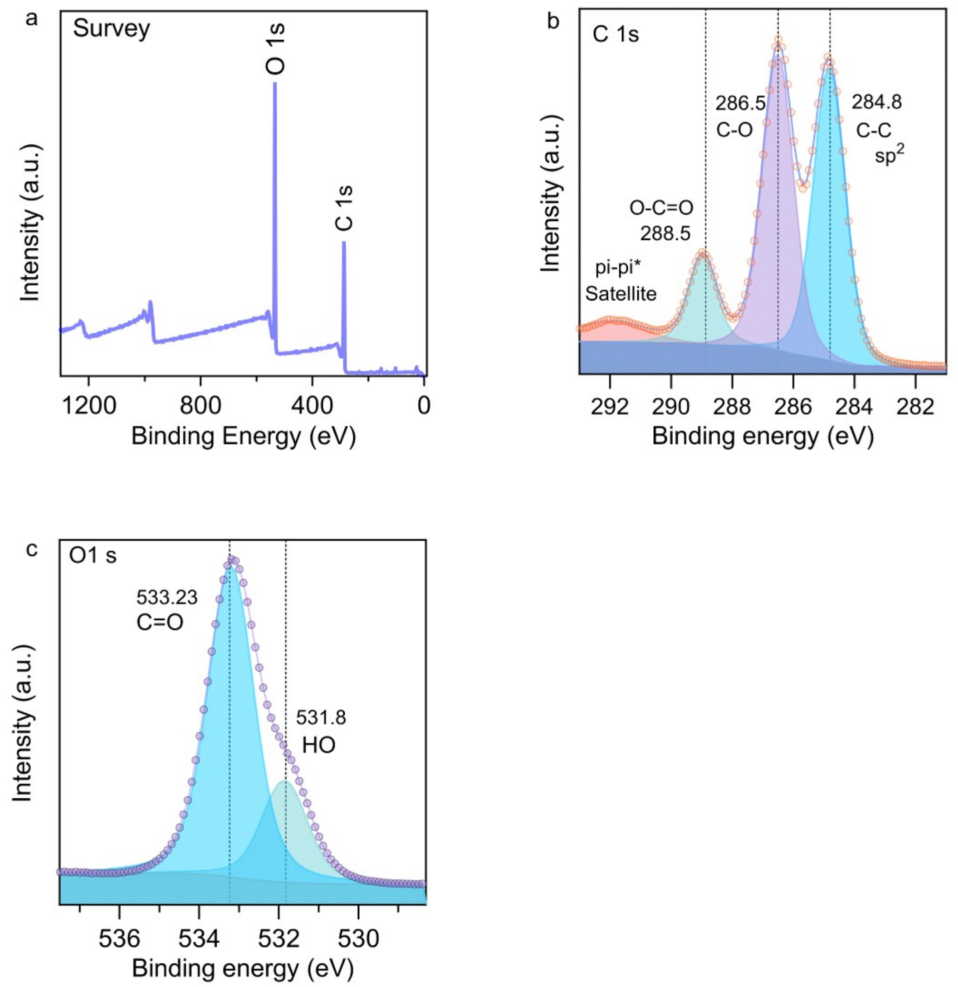

Figure S9. The XPS spectra of TA. (a) Survey spectra showing the presence of O 1s, C 1s, (b) C 1s core level spectra, (c) O 1s core level spectra.

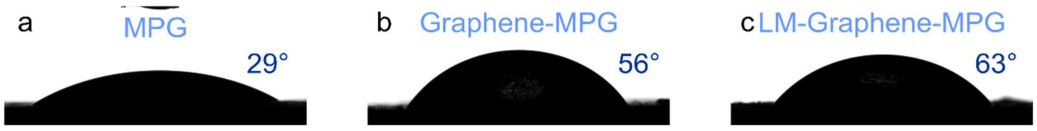

Figure S10. Water contact angles of (a) MPG, (b) Graphene–MPG, and (c) LM–Graphene–MPG.

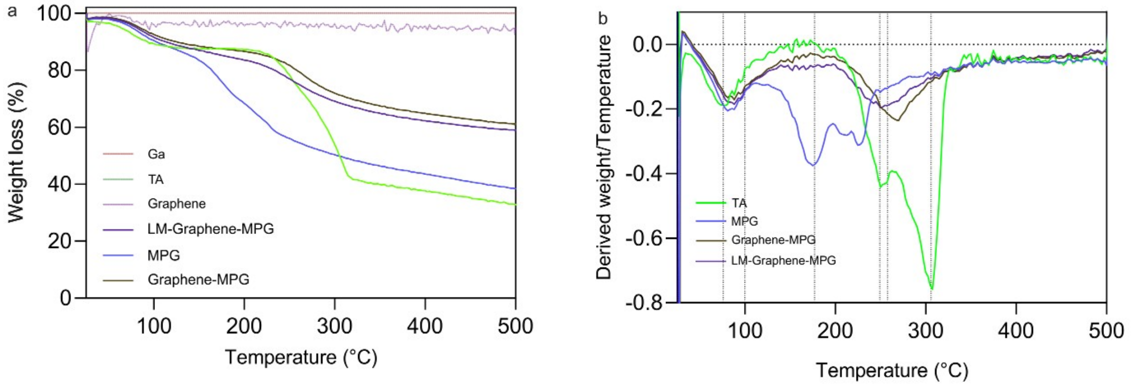

Figure S11. TG (a) and the first derivative of weight loss (DTG/(%/°C)) (b) of Ga, TA, Graphene, LM–Graphene–MPG, MPG, Graphene–MPG under nitrogen.

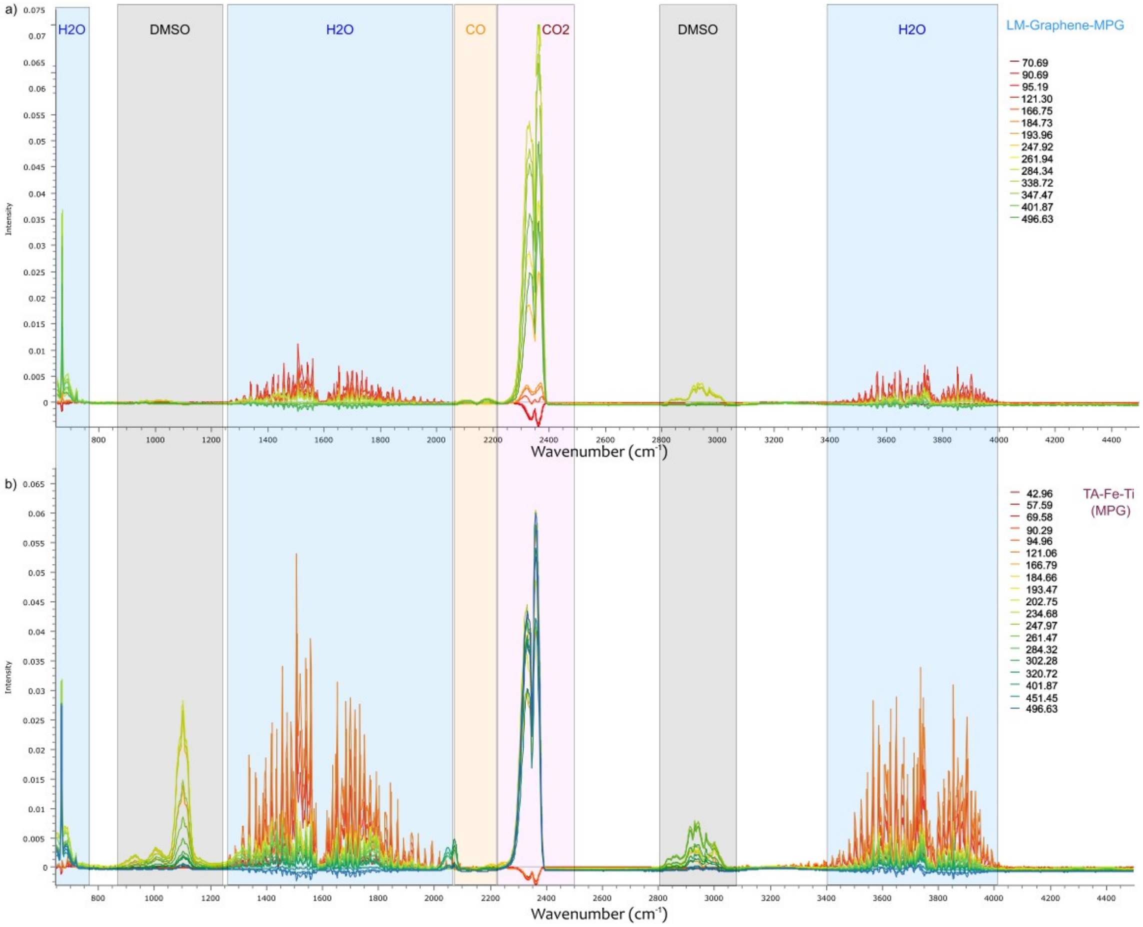

Figure S12. Evolution of degradation of products as a function of temperature. FTIR spectra of gaseous byproducts released during TGA at different temperatures under nitrogen for: (a) LM–Graphene–MPG (b) MPG.

The thermal degradation mechanism was clarified by TGA-FTIR spectroscopy, for LM–Graphene–MPG sample, the FTIR spectra captured from evolved gaseous species from thermal

degradation under N_2 atmosphere. During the thermal degradation process, the main volatilization products of are hydrocarbons (2949, 2876 and 2697 cm^{-1}), CO_2 (2359 and 2324 cm^{-1}), CO (2185 cm^{-1}), ester or ether components (1740 and 1105 cm^{-1}), aromatic compounds (1453 cm^{-1}), DMSO (3000, 1102 and 670 cm^{-1}), and water (4000-3500 cm^{-1} and 2000-1330 cm^{-1}) were identified in the gas phase in comparison with standard gas phase FTIR spectra from NIST Chemistry WebBook and Vapor phase library EPA-NIST.

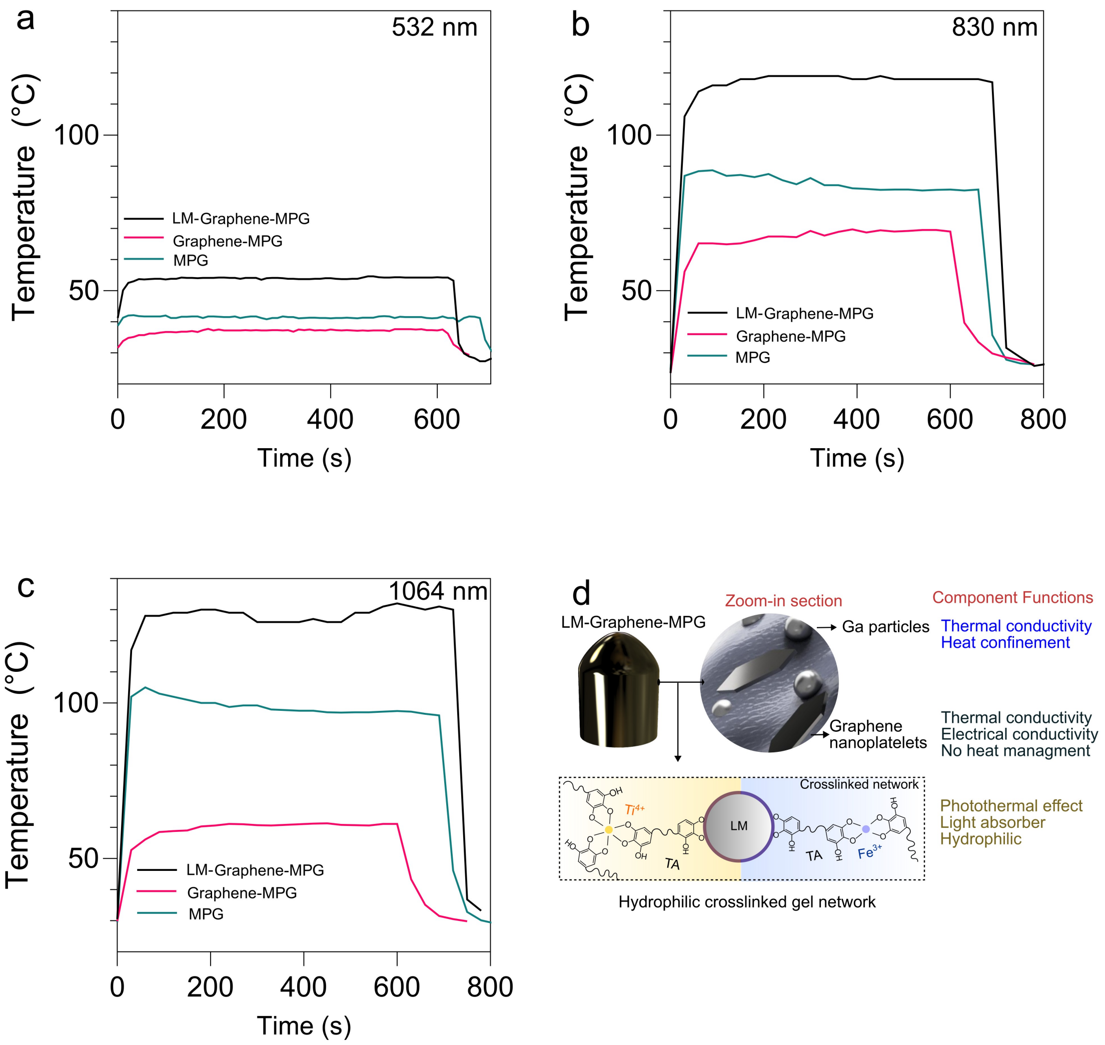

Figure S13. The IR thermal images under a) 532 nm (21.35 mW, 5.4 µm laser spot diameter, 9.32×10^4 W/cm^2, visible light). b) 830 nm (72.23 mW, 8.4 µm laser spot diameter, 1.30×10^5 W/cm^2, near-infrared irradiation), c)1064 nm (73 mW, 10.8 µm laser spot diameter, 7.97×10^4 W/cm^2, near-infrared irradiation). All images were captured using a 5x objective. d) Schematic illustration of the components functions of the LM–Graphene–MPG composite

Under 830 nm and 1064 nm irradiation, MPG shows superior photothermal performance compared to Graphene-MPG, and a similar trend is observed under 532 nm visible light. The main reason is that graphene alone tends to dissipate absorbed heat rapidly rather than confining it, whereas the LM–Graphene–MPG matrix stabilizes the absorbed energy and promotes localized heating.

Supplementary Note S1:

The photothermal conversion efficiency was determined[3] as follows:

Based on the total energy balance for the system:

$$\sum_i m_i c_{p,i} \frac{dT}{dt} = Q_s - Q_{loss} \tag{S1}$$

where m_i (g) and $C_{p,i}$ (J g^{-1} K^{-1}) are the mass and heat capacity of the sample (determined experimentally), respectively. Q_s is the photothermal heat energy input by irradiating NIR laser to samples, and Q_{loss} is the thermal energy lost to the surroundings. When the temperature is maximum, the system is in balance, where:

$$Q_s = Q_{loss} = hS\Delta T_{max} \tag{S2}$$

where h represents the heat transfer coefficient, S is the surface area of the specimen, $\Delta T_{(max)}$ is the maximum temperature variation.

The photothermal conversion efficiency η is calculated based on the following equation

$$\eta_s = \frac{hS\Delta T_{max}}{I(1-10^{-A_\lambda})} \tag{S3}$$

where I is the effective laser power (W), and A_λ is the absorbance of the absorber (LM–Graphene–MPG) at the wavelength of λ nm.

In order to obtain the *hS*, a dimensionless driving force temperature, θ is introduced as follows:

$$\theta = \frac{T - T_{surr}}{T_{max} - T_{surr}} \tag{S4}$$

Where T is the temperature of the sample, T_{max} is the maximum temperature (°C), and T_{surr} is the initial temperature (°C).

The time constant τ_s for the sample system:

$$\tau_s = \frac{\sum_i m_i c_{p,i}}{hS} \tag{S5}$$

Thus $\frac{d\theta}{dt} = \frac{1}{\tau_s}\frac{Q_s}{hS\Delta T_{max}} - \frac{\theta}{\tau_s}$

When the laser is off, Q_s= 0, therefore $\frac{d\theta}{dt} = -\frac{\theta}{\tau_s}$, and $t = -\tau_s ln\theta$

So *hS* could be calculated from the slope of cooling time vs ln θ plot. (**Figure S14**).

Table S1. Photothermal efficiency for 532nm, 830 nm, 1064 nm

Samples	τs [slope] (s)	Tmax (°C)	Tsurr (°C)	ΔT (K)	Mass (g)	$c_{p,i}$ ($Jg^{-1} K^{-1}$)	I power (W)	Absorbance	η
**532 nm**									
LM-Graphene-MPG	14.042	48.78	26.20	22.58	0.0139	0.8456	0.02135	0.968	**0.99**
**830 nm**									
LM-Graphene-MPG	14.232	50.46	27.29	23.17	0.0139	1.0587	0.03613	0.958	**0.75**
**1064 nm**									
LM-Graphene-MPG	24.751	59.10	24.54	34.56	0.0139	1.1218	0.0365	0.954	**0.67**

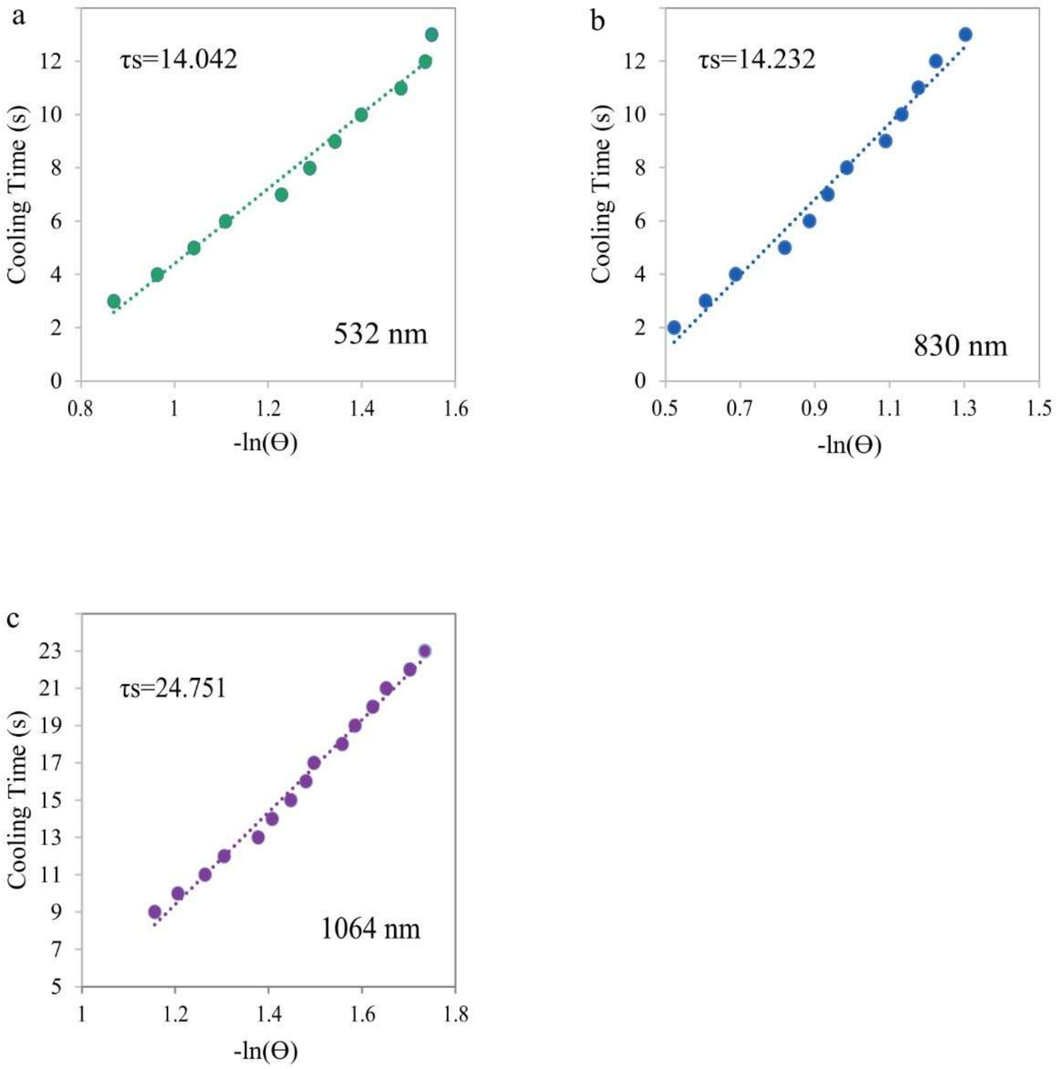

Figure S14. Cooling time versus -ln(θ) linear curve for the η calculation.

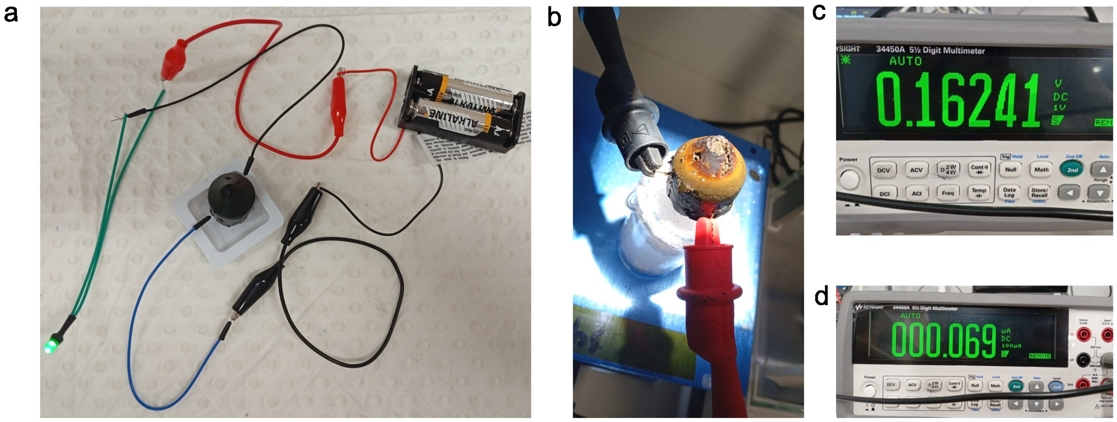

Figure S15. a) Experimental setup used for the electrical conductivity measurement for the LM–Graphene–MPG. b) Photographs of LM–Graphene–MPG under one sun during solar steam generation of seawater. c) Open-circuit voltage measurement. d) Short-circuit current measurement of a single LM–Graphene–MPG.

Table S2. Measured resistance and calculated electrical conductivity of samples.

Sample	Resistance (Ω)	Electrical conductivity (S/m)
LM–Graphene–MPG	10.606±0.508	1118.04
Graphene–MPG	2.379±0.046	83784.50
MPG	10241093±6476449	0.02

Equation for the calculation of the electrical conductivity of the samples

$$\sigma = \frac{w}{R \times l \times d} \qquad \text{(S6)}$$

σ: electrical conductivity
R: resistance
d: thickness
l: length
w: width

By a 2-point probe method, the room temperature conductivity (σ) was measured.

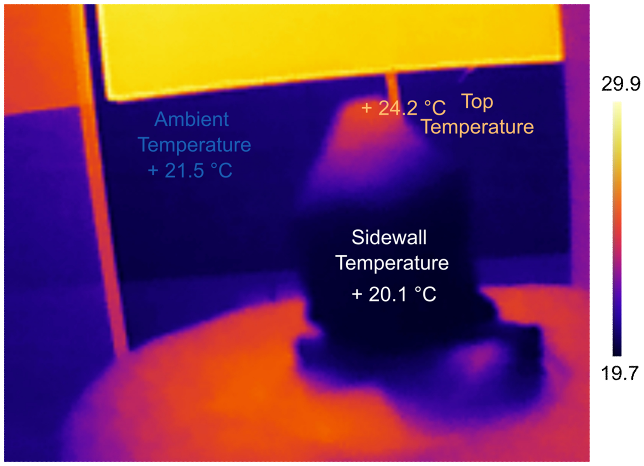

Figure S16. Infrared image of the side-view of LM-Graphene-MPG under 1 sun irradiation.

Supplementary Note S2: Heat loss analysis

1. Energy Input

The system was tested using a solar simulator calibrated to 1 sun (1000 W/m^2) under ambient temperature (22.5 °C) with 35% of relative humidity in minimal airflow conditions.

The solar power input per unit area (E_{solar}) is calculated as:

$$E_{solar} = C_{opt} P_o A_{proj} \tag{S7}$$

Where C_{opt} is the optical concentration factor (1 in our case), and P_o is the solar radiation power for one sunlight intensity 1000 W/m^2, and A_{proj} is 0.000564 m^2

$$E_{solar} = (1)x(1000\, W/m^2)\; x\; 0.000564\ \text{m}^2 = 0.564\text{W} = 564\ \text{mW}$$

2. Energy loss analysis

The total energy input is then converted into useful energy (for evaporation) and various forms of heat loss. The heat loss by absorber consists of four parts: reflection, radiation, convection, conduction.

2.1. Reflected solar light on the surface

The measured average reflection loss of LM-Graphene-MPG over the broad solar spectrum (200-2500 nm) is 4.3 %.

2.2. Radiative losses

The radiation loss was calculated by Stefan-Boltzmann formula.

$$Q_{rad-top} = \epsilon \sigma A_{top}\left({T_{top}}^4 - T_{amb}^4\right) \tag{S8-9}$$

$Q_{rad-side} = \epsilon\sigma A_{side}\left(T_{side}{}^{4} - T_{amb}^{4}\right)$

where A_{top} is the area of top surface (0.000789 m^2), ε is the emissivity of the evaporator surface (0.9404), σ is the Stefan-Boltzmann constant (5.6703 × 10^{-8} W m^{-2} K^{-4}), T_{top} is the top temperature of the evaporator (297.3 K), T_{side} is the average temperature of the side surface (294.65 K), T_{amb} is the near-surface ambient temperature (295.35 K).

Heat radiation losses are estimated at $Q_{rad-top}$= 8.54 mW (1.51%), $Q_{rad-side}$= -4.94 mW (**gain of energy 0.87%)**

2.3. Convective losses

The convection heat loss is caused by air flow, which can be analysed by Newton's law of cooling.

$$Q_{conv-top} = A_{top}h_c\left(T_{top} - T_{amb}\right)$$

$$Q_{conv-side} = A_{sideh_c}(T_{side} - T_{amb}) \qquad \text{(S10-11)}$$

Where A_{top} is the area of top surface (0.000789 cm^2), A_{side} is the effective area of side surface for convective heat transfer (0.00129 cm^2), h is the convention heat transfer coefficient (~5 W $m^{-2}K^{-1}$), T_{top} is the steady-state surface temperature of the evaporator top (297.3 K), T_{side} is the steady-state surface temperature of the evaporator side surface (294.65 K) and T_{amb} is the near-surface ambient temperature (295.35 K).

Heat convection losses are estimated at $Q_{conv-top}$ 12.58 mW (2.23%), $Q_{conv-side}$= -4.52 mW (**gain of energy 0.80%)**

2.4. Conduction losses

To calculate the conduction loss, the 3D is put on the thermally isolated water container from surroundings. The conductive heat dissipation to bulk water from evaporator according to the following equation.

$$Q_{cond} = Cm\Delta T \qquad \text{(S12)}$$

Where C is the specific heat capacity of water (4.2 J/gK), m is the weight of bulk water (26.3514 g) and ΔT represents the increased temperature of bulk water measured at steady state exhibiting stable steam generation rates, which is mostly achieved within 3600 s under irradiation (2 K).

Q_{cond}= 221.35 J, W_{solar}= P_{solar}time = 0.564W x 3600 s=2030.4 J, 10.9%

Therefore, the sum of calculated energy losses is 18.94 % under 1 sun illumination.

Total Energy gain from ambient is 1.68%

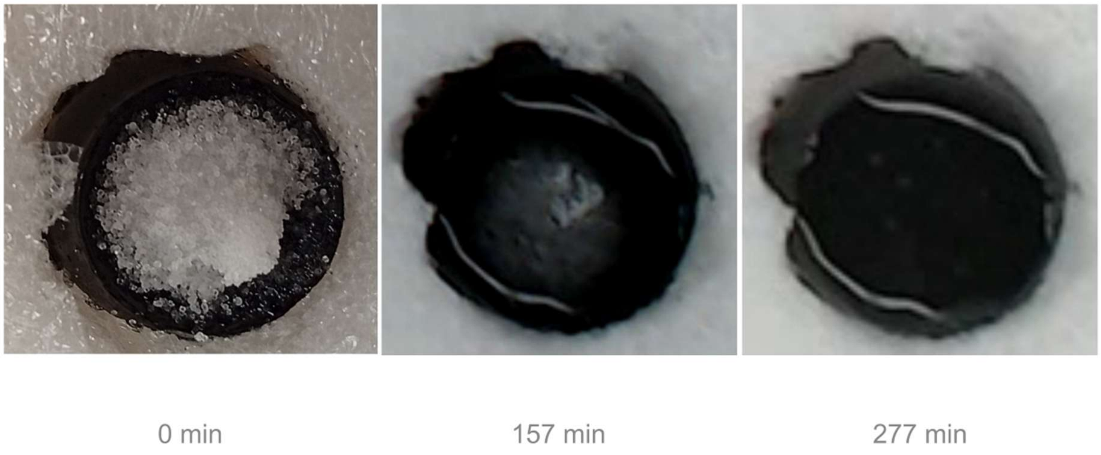

Figure S17. Self-cleaning process and salt antifouling performance of MPG. Sequential photographs of MPG with 0.5 g of NaCl placed on its surface, demonstrating salt dissolution and removal over time. The gel is floating on artificial seawater (3.5 wt% NaCl) under dark conditions.

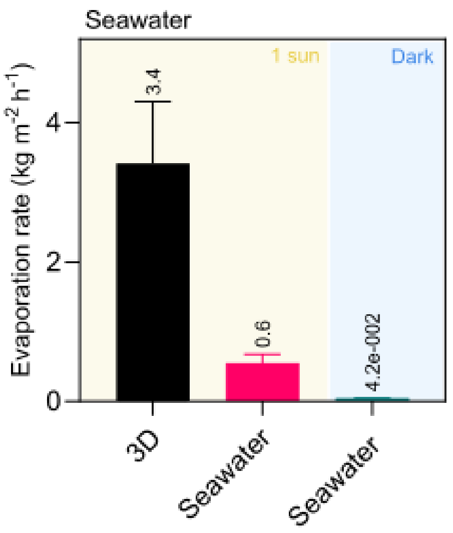

Figure S18. Evaporation rate of LM–Graphene–MPG in seawater under one sun illumination.

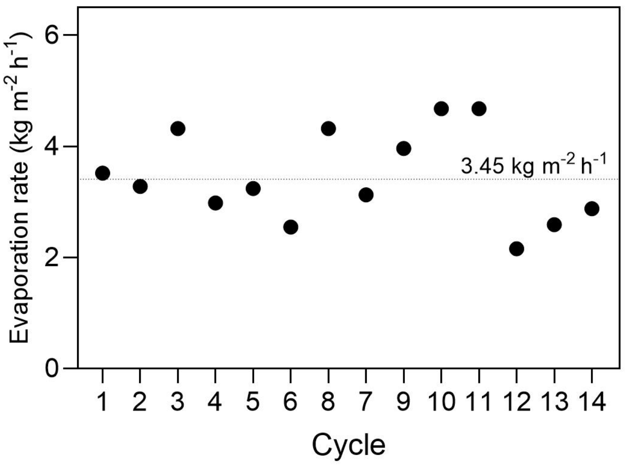

Figure S19. Stability tests for LM–Graphene–MPG for seawater evaporation over 14 cycles (1 h for each cycle). Between cycles the LM–Graphene–MPG was immersed in seawater.

Table S3. Evaporation rate compared with references

	Material category	Evaporation rate (kg $m^{-2}h^{-1}$)	Water type	Solar Thermal conversion efficiency (%)	Surface configuration	Reference
PCH-Chitosan Gel-Hukaiwen Ink	Hydrogel/Carbon Ink	3.3	Natural wastewater		2D	[4]
Agar Gel/TiN	Hydrogel/ Organic Absorber	5.15	Water		2D	[5]
PAA/PEDOT	Hydrogel/Inorganic Absorber	3.05	Water	95	2D	[6]
V-rGO Foam	Synthetic foam	3.39	Water	104.1	3D	[7]
PVA/PPy	Hydrogel/Organic Absorber	3.6	Water	96	3D	[8]
EGaInLMTE PVA/PAM	Hydrogel/ metal particles	2.96	Water	96.93	3D	[9]
Ag@C/PVA/LM-GAs-Fe^{III}	Gel/Carbon/metal particles	3.23	Water	93.1	2D	[10]
PVA/PM/C-EGaInP-3	Hydrogel/ metal particles	1.52	Water		2D	[11]
L-CNC/STA EGaIn	Aerogel/ metal particles	1.38	Water	94	2D	[12]
EGaIn@Ag/PVAG	Hydrogel/ metal particles	3.26	Water		2D	[13]
Ga@EOG/PVA	Aerogel/ metal particles	5.13	Water	98.2	3D	[14]
LLM/DW	Biomass/metal particles	2.23	Water	95.9	2D	[15]
**Our work**	Gel/Carbon/metal particles	4.8	Water		3D	
PVA/graphene Oxide Hydrogel	Hydrogel/Inorganic Absorber	3.15	Seawater	99.98	2D	[16]
PVA/PPy	Hydrogel/Organic Absorber	3.2	Seawater	94	2D	[17]
Balsa Wood	Biomass	3.91	Seawater		3D	[18]
Carbon black PVA-SO_4^{2-}	Hydrogel/Carbon	3.52	Seawater	97.2	3D	[19]
PPC/polyacrylamide	Hydrogel	4.85	Seawater		3D	[20]
**Our work**	Gel/Carbon/metal particles	3.4	Seawater		3D	
Graphene oxides upon porous melamine sponge	Carbon-based	3.47	Simulated Seawater	97.4		[21]
LLM/DW	Metal particles	1.90	Simulated Seawater		2D	[15]
Nitrogen-doped CNTs encapsulated with Co/carbon fibers	Hybrid	3.85	Simulated Seawater	98	3D	o
Carbon nanofiber/graphene oxide composite	Aerogel/Carbon	3.47	Simulated Seawater	97.5	3D	[22]
MXene/polydopamine (PDA)	Hydrogel/Inorganic Absorber	3.02	Simulated seawater	94.7	2D	[23]

Mxene-rGO	Sponge/Carbon	2.35	Simulated Seawater	127	2D	[24]
Sodium Alginate/Polyamine/Carbon Nanotubes	Inorganic Hydrogel/Polymer/Absorber/Carbon	3.2	Simulated Seawater	95	3D	[25]
Ag@C/PVA/LM-GAs-Fe^{III}	Gel/Carbon/metal particles	3.47	Simulated Seawater		2D	[10]
**Our work**	Gel/Carbon/metal particles	4.0	Simulated Seawater		3D	

Simulated Seawater 3.5% w/w NaCl

Table S4: Abbreviations

N-CNTs@Co/C	Nitrogen-doped CNTs encapsulated with Co/carbon fibers
GO@melamine	Graphene oxides upon porous melamine sponge
C nanofiber/GO	Carbon nanofiber/graphene oxide composite
PVA/GO gel	Polyvinyl Alcohol/ graphene Oxide gel
Salg/poly/CNTs	Sodium Alginate/Polyamine/Carbon Nanotubes
Mxene/rGO	MXene-reduced graphene oxide sponge-
Agar gel/TiN	Agar and Titanium nitride hydrogel based evaporators
PVA/PPY	Polyvinyl alcohol/Polypyrrole hydrogel
V/rGO	Vertically aligned reduced graphene oxide foam
PAA/PEDOT	poly (acrylic acid)/poly(3,4-ethylene dioxythiophene) hydrogel
PVA/PM/C-EGaInP-3	Polyvinyl alcohol/poly(acrylamide)/Cellulose nanocrystals EGaIn/polyaniline complex
L-CNC/STA EGaIn	Lignin cellulose nanocrystal aerogel/Stearic acid-EGaIn
Ag@C/PVA/LMGAs-Fe^{III}	Carbon wrapped silver nanowire sponge/ Polyvinyl alcohol/EGaIn/Galic acid iron
3DPxLMTE@ MPN	EGaIn Tannic acid Metal Phenolic Network Hydrogel
EGaIn@Ag/PVAG	EGaIn Hydrogel
Ga@EOG/PVA	Gallium Edge Oxidized Graphene polyvinyl alcohol with vertical pore structure
LLM/DW	Lignin EGaIn/Delignified wood
PMMA	Poly(methyl methacrylate)

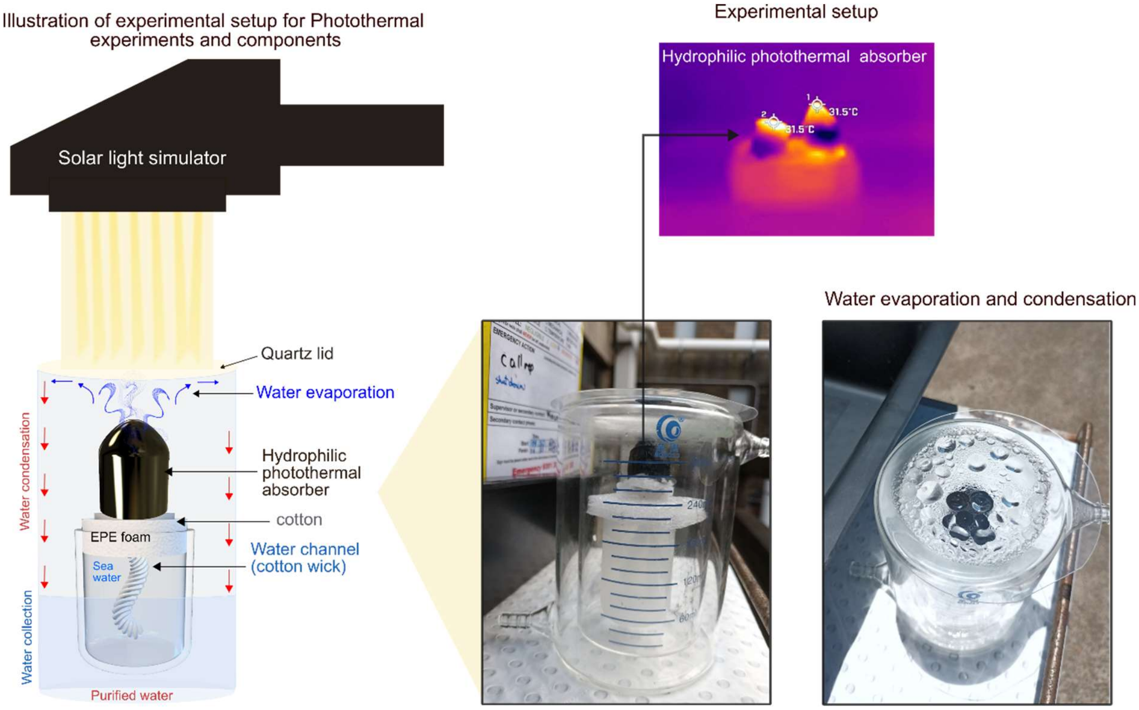

Figure S20. Illustration of the experimental setup used for solar steam generation and water collection.

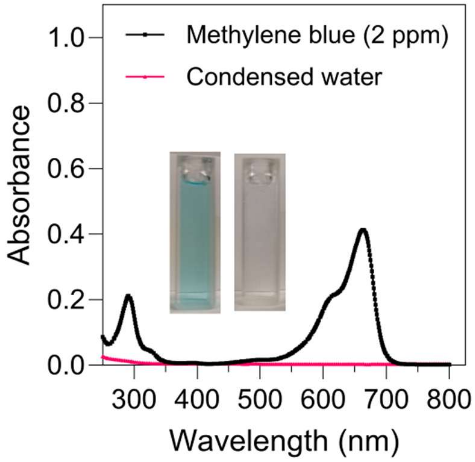

Figure S21. UV-vis spectra and photograph of dye contaminated water and purified water obtained from using LM–Graphene–MPG evaporator.

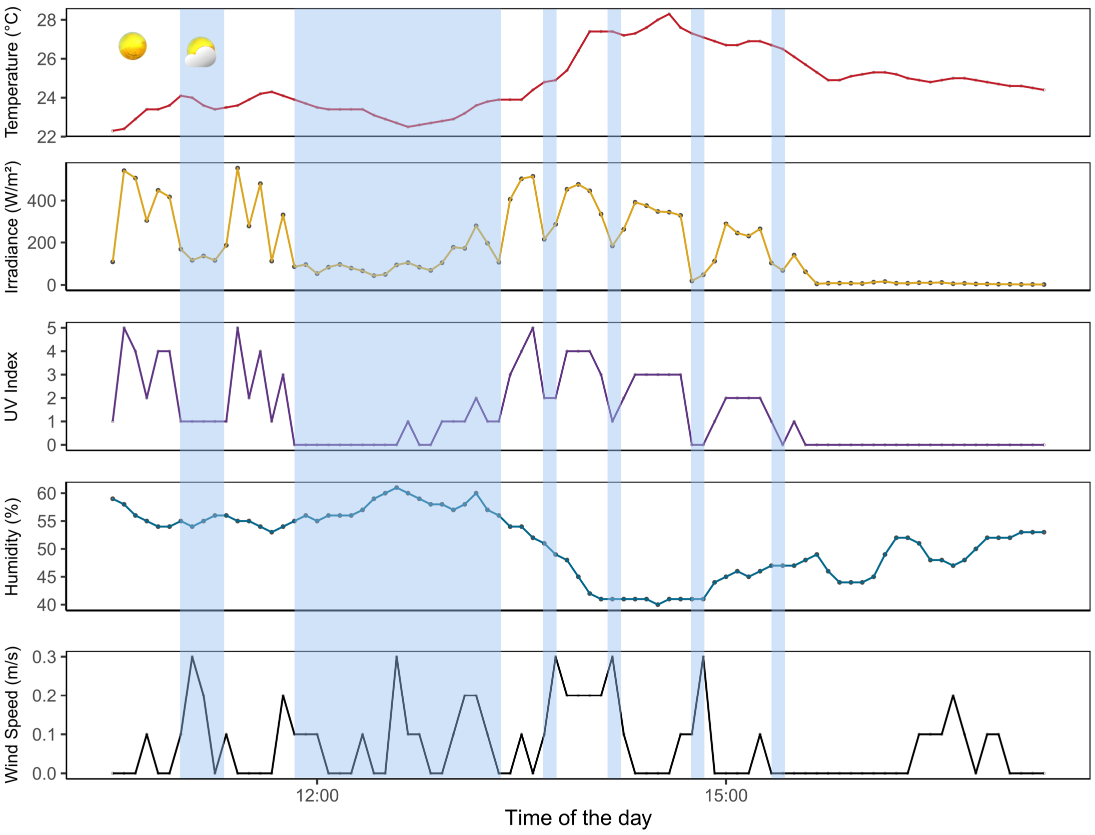

Figure S22. Changes in temperature, outdoor solar radiation, UV index, humidity and wind speed during outdoor experiment (10:25-17:25 09/09/25 Sydney, Australia).

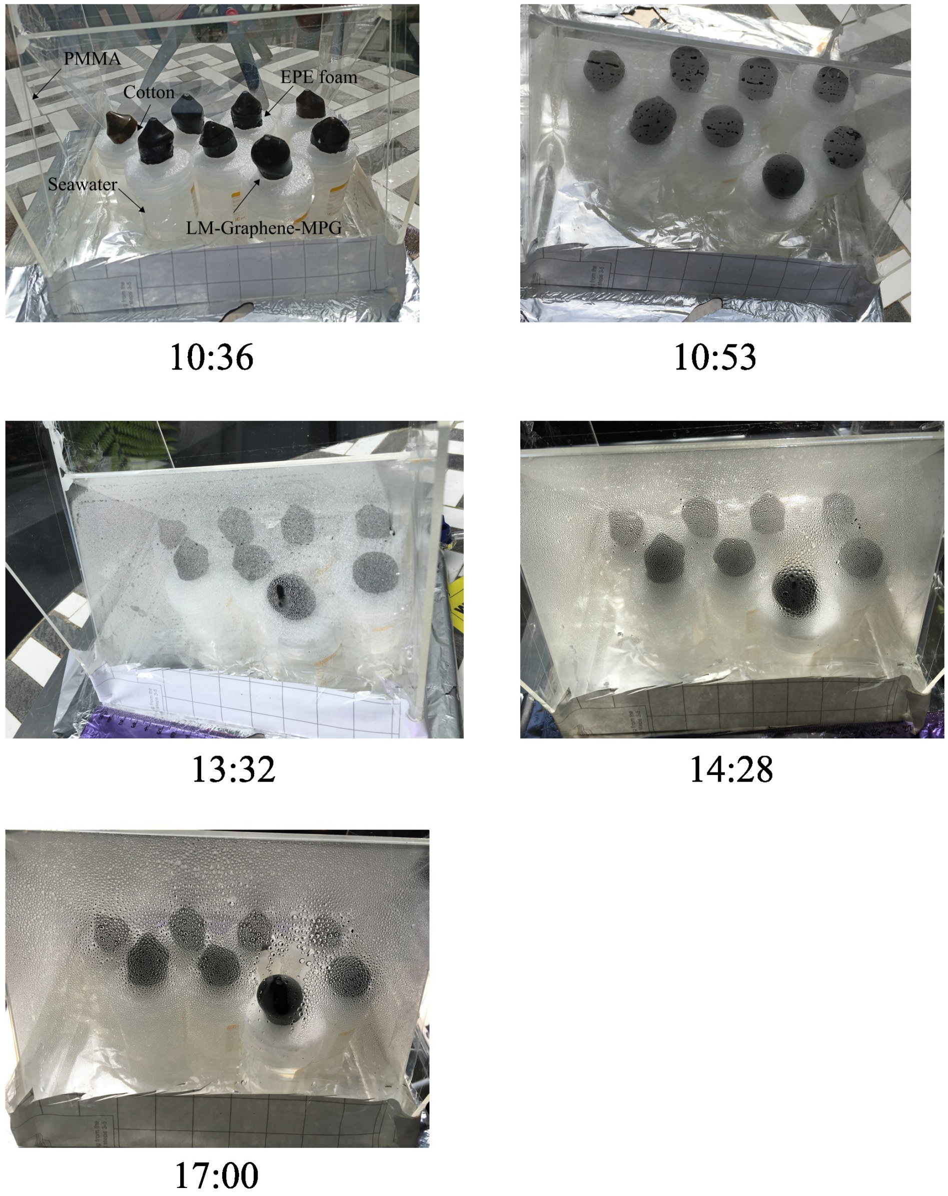

Figure S23. Photograph of outdoor experiment for water purification of seawater from Maroubra beach from 10:25-17:25 09/09/25 Sydney, Australia.

Table S5. Material cost for LM-Graphene-MPG preparation

Material	Typical Supplier Form	Approx. Cost (USD)	Price sources
Graphene nanoplatelets (5 µm)	Powder	$ 127.15/250 g	https://www.sigmaaldrich.com/AU/en/product/aldrich/900412
MilliQ Water	Liquid		
LM (Gallium)	Solid	$ 688.85/500 g	https://www.rotometals.com/gallium/
Tannic acid	Solid	$ 138.83/500g	https://www.thermofisher.com/order/catalog/product/202425000
Isopropanol	Liquid	$ 19.76/5 L	https://www.amazon.com.au/Isopropyl-Alcohol-Industrial-Multi-Purpose-Fast-Drying/dp/B0F4JZGCKM/ref
MilliQ Water	Liquid		
Titanium (IV) bis(ammonium lactato)dihydroxide solution	Liquid	$ 42.69/100 ml	https://www.sigmaaldrich.com/AU/en/product/aldrich/388165
Dimethyl sulfoxide	Liquid	$ 53.63/500ml	https://shop.chemsupply.com.au/dimethyl-sulfoxide-ar-dmso
Iron (III) nitrate nonahydrate	Solid	$ 49.87/100g	https://www.sigmaaldrich.com/AU/en/product/sigald/216828

Table S6. The LM-Graphene-MPG can be translated to:

Material	For 1 sample	Cost ($)
Graphene nanoplatelets	150 mg	0.07629
Water	7.5 ml	
LM (Gallium)	609.5 mg	0.419854
Tannic acid	1.1215 g	0.311401
Isopropanol	2.1155 g	0.010637
Water	1.6448 g	
Titanium (IV) bis (ammonium lactato) dihydroxide solution	0.75 ml	0.320175
Iron (III) nitrate nonahydrate	500 mg	0.24935
Total cost/ per sample		**1.47 USD**

Based on laboratory-scale fabrication, the estimated cost of our evaporator system (area 6.249 cm^2) was estimated to be approximately $ 0.24/cm^2.

References

[1] M. J. Sever, J. J. Wilker, *Dalton Trans.* **2004**, DOI: 10.1039/B315811J1061.
[2] B. A. Borgias, S. R. Cooper, Y. B. Koh, K. N. Raymond, *Inorg. Chem.* **1984**, 23, 1009.
[3] X. Huang, J. Liu, P. Zhou, G. Su, T. Zhou, X. Zhang, C. Zhang, *Small* **2022**, 18, 2104048.
[4] Q. Xiao, Y. Zhu, Y. Xi, X. Kong, X. Ye, Z. Zhang, C. Qiu, W. Xu, S. Cheng, J. Zhang, M. Jia, E. Sun, H. Lin, J. Wang, *Chem. Eng. J.* **2022**, 430, 133019.
[5] Y. Tian, X. Liu, S. Xu, J. Li, A. Caratenuto, Y. Mu, Z. Wang, F. Chen, R. Yang, J. Liu, M. L. Minus, Y. Zheng, *Desalination* **2022**, 523, 115449.
[6] Y. Peng, S. Tang, X. Wang, R. Ran, *Macromol. Mater. Eng.* **2021**, 306, 2100309.
[7] W. Li, X. Tian, X. Li, S. Han, C. Li, X.-Z. Zhai, Y. Kang, Z.-Z. Yu, *J. Mater. Chem. A.* **2021**, 9, 14859.
[8] Y. Shi, O. Ilic, H. A. Atwater, J. R. Greer, *Nat. Commun.* **2021**, 12, 2797
[9] S. Yang, H. Zhang, X. Sun, J. Bai, J. Zhang, *ACS Nano* **2024**, 18, 5847.
[10] S. Yang, Y. He, J. Bai, J. Zhang, *Small* **2023**, 19, 2302526.
[11] Z. Wei, Y. Wang, C. Cai, Y. Zhang, S. Guo, Y. Fu, S. C. Tan, *Adv. Funct. Mater.* **2022**, 32, 2206287.
[12] Z. Wei, C. Cai, Y. Huang, Y. Wang, Y. Fu, *Nano Energy* **2021**, 86, 106138.
[13] F. Yang, Z. Bao, Z. Liang, G. He, J. Li, Q. Liang, J. Li, S. Luo, Y. Liu, *Chem. Eng. J.* **2024**, 490, 151815.
[14] X.-Z. Zhang, D. Wu, Y.-H. Yang, D. Xiang, Y.-L. Zhu, E. Harkin-Jones, Y.-P. Wu, Q. Fu, Z.-K. Yan, H. Deng, *Rare Met.* **2025**, 44, 4038.
[15] Y. Chen, R. Liu, R. Cao, A. Nilghaz, X. Wan, G. Chen, J. Tian, *Compos. B: Eng.* **2025**, 302, 112543.
[16] Y. Chen, X. Zhao, Z. Ye, Y. Chen, P. Lin, *Desalination* **2022**, 522, 115406.
[17] F. Zhao, X. Zhou, Y. Shi, X. Qian, M. Alexander, X. Zhao, S. Mendez, R. Yang, L. Qu, G. Yu, *Nature Nanotechnology* **2018**, 13, 489.
[18] J. Tang, T. Zheng, Z. Song, Y. Shao, N. Li, K. Jia, Y. Tian, Q. Song, H. Liu, G. Xue, *ACS Appl. Mater. Interfaces.* **2020**, 12, 18504.
[19] J. Ren, L. Chen, J. Gong, J. Qu, R. Niu, *Chem. Eng. J.* **2023**, 458, 141511.
[20] J. Zhou, Z. Sun, X. Mu, J. Zhang, P. Wang, Y. Chen, X. Wang, J. Gao, L. Miao, L. Sun, *Desalination* **2022**, 537, 115872.
[21] J. Su, Q. Chang, C. Xue, J. Yang, S. Hu, *Carbon* **2022**, 194, 267.
[22] L.-B. Zhong, S.-J. Chen, X. Hou, Q.-J. Zhang, C.-Y. Guo, Y.-M. Zheng, *Sci. China Mater.* **2023**, 66, 3300.
[23] M. Pi, X. Wang, Z. Wang, R. Ran, *Polymer* **2021**, 230, 124075.
[24] Z. Liu, R. Ding, F. Xue, X. Zhao, Z. Chen, H. Zheng, P. Li, Q. Yan, L. Xu, J. Xiong, Q. Peng, X. He, *Commun. Mater.* **2023**, 4, 70.
[25] C. Liu, Y. Peng, X. Zhao, *Carbohydr. Polym.* **2021**, 273, 118536.
